# Experimental and computational modeling for signature and biomarker discovery of renal cell carcinoma progression

**DOI:** 10.1186/s12943-021-01416-5

**Published:** 2021-10-20

**Authors:** Lindsay S. Cooley, Justine Rudewicz, Wilfried Souleyreau, Andrea Emanuelli, Arturo Alvarez-Arenas, Kim Clarke, Francesco Falciani, Maeva Dufies, Diether Lambrechts, Elodie Modave, Domitille Chalopin-Fillot, Raphael Pineau, Damien Ambrosetti, Jean-Christophe Bernhard, Alain Ravaud, Sylvie Négrier, Jean-Marc Ferrero, Gilles Pagès, Sebastien Benzekry, Macha Nikolski, Andreas Bikfalvi

**Affiliations:** 1 University of Bordeaux, LAMC, Pessac, France; 2 INSERM U1029, Pessac, France; 3 Bordeaux Bioinformatics Center, CBiB, University of Bordeaux, Bordeaux, France; 4 Mathematical Modeling for Oncology Team, Inria Bordeaux Sud-Ouest, Talence, France; 5 Department of Mathematics, Mathematical Oncology Laboratory (MOLAB), Universidad de Castilla-La Mancha, Ciudad Real, Spain; 6 University of Liverpool, Institute of Systems, Molecular and Integrative Biology, Liverpool, UK; 7 Centre Scientifique de Monaco, Biomedical Department, Principality of Monaco, Monaco; 8 University Côte d’Azur, Institute for Research on Cancer and Aging of Nice (IRCAN), CNRS UMR 7284; INSERM U1081, Centre Antoine Lacassagne, Nice, France; 9 VIB-KU Leuven Center for Cancer Biology, Leuven, Belgium; 10 University of Bordeaux, IBGC, Bordeaux, France; 11 University of Bordeaux, “Service Commun des Animaleries”, Bordeaux, France; 12 Centre Hospitalier Universitaire (CHU) de Nice, Hôpital Pasteur, Central laboratory of Pathology, Nice, France; 13 Centre Hospitalier Universitaire (CHU) de Bordeaux, service d’urologie, Bordeaux, France; 14 Centre Hospitalier Universitaire (CHU) de Bordeaux, service d’oncologie médicale, Bordeaux, France; 15 Université de Lyon, Centre Léon Bérard, Lyon, France; 16 Centre Antoine Lacassagne, Clinical Research Department, Nice, France; 17 COMPO team-project, Inria Sophia Antipolis and CRCM, Inserm U1068, CNRS UMR7258, Aix-Marseille University UM105, Institut Paoli-Calmettes, Marseille, France

**Keywords:** Metastasis, Prognostic markers renal cell carcinoma, Systems biology approach, Tumor model, SAA2, CFB, Computational model

## Abstract

**Background:**

Renal Cell Carcinoma (RCC) is difficult to treat with 5-year survival rate of 10% in metastatic patients. Main reasons of therapy failure are lack of validated biomarkers and scarce knowledge of the biological processes occurring during RCC progression. Thus, the investigation of mechanisms regulating RCC progression is fundamental to improve RCC therapy.

**Methods:**

In order to identify molecular markers and gene processes involved in the steps of RCC progression, we generated several cell lines of higher aggressiveness by serially passaging mouse renal cancer RENCA cells in mice and, concomitantly, performed functional genomics analysis of the cells. Multiple cell lines depicting the major steps of tumor progression (including primary tumor growth, survival in the blood circulation and metastatic spread) were generated and analyzed by large-scale transcriptome, genome and methylome analyses. Furthermore, we performed clinical correlations of our datasets. Finally we conducted a computational analysis for predicting the time to relapse based on our molecular data.

**Results:**

Through in vivo passaging, RENCA cells showed increased aggressiveness by reducing mice survival, enhancing primary tumor growth and lung metastases formation. In addition, transcriptome and methylome analyses showed distinct clustering of the cell lines without genomic variation. Distinct signatures of tumor aggressiveness were revealed and validated in different patient cohorts. In particular, we identified SAA2 and CFB as soluble prognostic and predictive biomarkers of the therapeutic response. Machine learning and mathematical modeling confirmed the importance of CFB and SAA2 together, which had the highest impact on distant metastasis-free survival. From these data sets, a computational model predicting tumor progression and relapse was developed and validated. These results are of great translational significance.

**Conclusion:**

A combination of experimental and mathematical modeling was able to generate meaningful data for the prediction of the clinical evolution of RCC.

**Supplementary Information:**

The online version contains supplementary material available at 10.1186/s12943-021-01416-5.

## One sentence summary

An aggressiveness screen with multilayer systems analysis to identify signatures and biomarkers for renal cell carcinoma aggressiveness.

## Introduction

Renal Cell Carcinoma (RCC) encompasses a heterogeneous group of cancers derived from renal tubular epithelial cells, including multiple histological and molecular sub-types, of which clear cell RCC (ccRCC) is the most common [1]. RCC accounted for around 179,000 worldwide deaths during the last year, and its mortality is predicted to double in the next 20 years (Globocan project 2020, update December 2020 [2]). The major issue for RCC patients is the absence of an efficient therapeutic option, especially for recurrent and metastatic forms of the disease. Localized RCC is treated by surgical resection and has a good 5-year survival rate. However, 40% of patients with seemingly localized disease later relapse with localized or metastatic RCC. Both localized recurrence and RCC metastasis are difficult to treat and give a poor prognosis [3]. The preferred therapeutic options for RCC treatment aim to target or tumor angiogenesis (i.e. Sunitinib or Bevacizumab, blockers of VEGF/VEGFR) [1] or the immune system (i.e. Ipilimumab + Nivolumab, two inhibitors of CTLA-4 and PD-1 respectively) [4–7]. However, such therapies are rarely curative, and drug resistance is almost inevitable. Furthermore, clinical treatment of RCC is hampered by a lack of relevant biomarkers. Patient diagnosis, prognosis and clinical decisions are currently based on histological information (i.e. Fuhrman grade or MSKCC score and tumor stage [8, 9], and therapy selection is based on limited guidelines and response to previous treatments. In this respect, clinical treatment of RCC lags behind other cancers for which molecular knowledge is invaluable in guiding clinical decisions (e.g. hormone receptors or human epidermal growth factor receptor 2 (HER2) status in breast cancer). In fact, little is known about the pathophysiological mechanisms of RCC initiation and progression. The elucidation of such mechanisms is essential to undertake efficient therapeutic interventions for RCC, especially for its metastatic forms.

Tumor progression from initiation to full metastasis is a multi-step process which occurs via a series of overlapping stages. Tumor progression can be seen as an evolutionary process whereby tumor cells must detach from the primary tumor, gain access to and survive in the circulation, exit the vasculature, and survive and proliferate in the environment of the secondary organ. Thus, different mechanisms come into play at different stages, and overall tumor progression is the sum of these processes [10]. A better understanding of the molecular changes occurring in the cancer cell during tumor progression steps could aid in the diagnosis, prevention and treatment of metastatic cancer, including RCC.

In this study, we generated increasingly aggressive renal cancer sub-lines by an in vivo serial implantation technique. Following this approach, we performed a detailed investigation associating functional genomics, an experimental and clinical validation of molecular signatures, and mathematical modelling to generate a set of meaningful results for a better understanding of RCC pathobiology and the clinic.

## Results

### Generation of enhanced aggressiveness renal cancer cell lines using a syngeneic mouse model of RCC

**Fig. 1:**
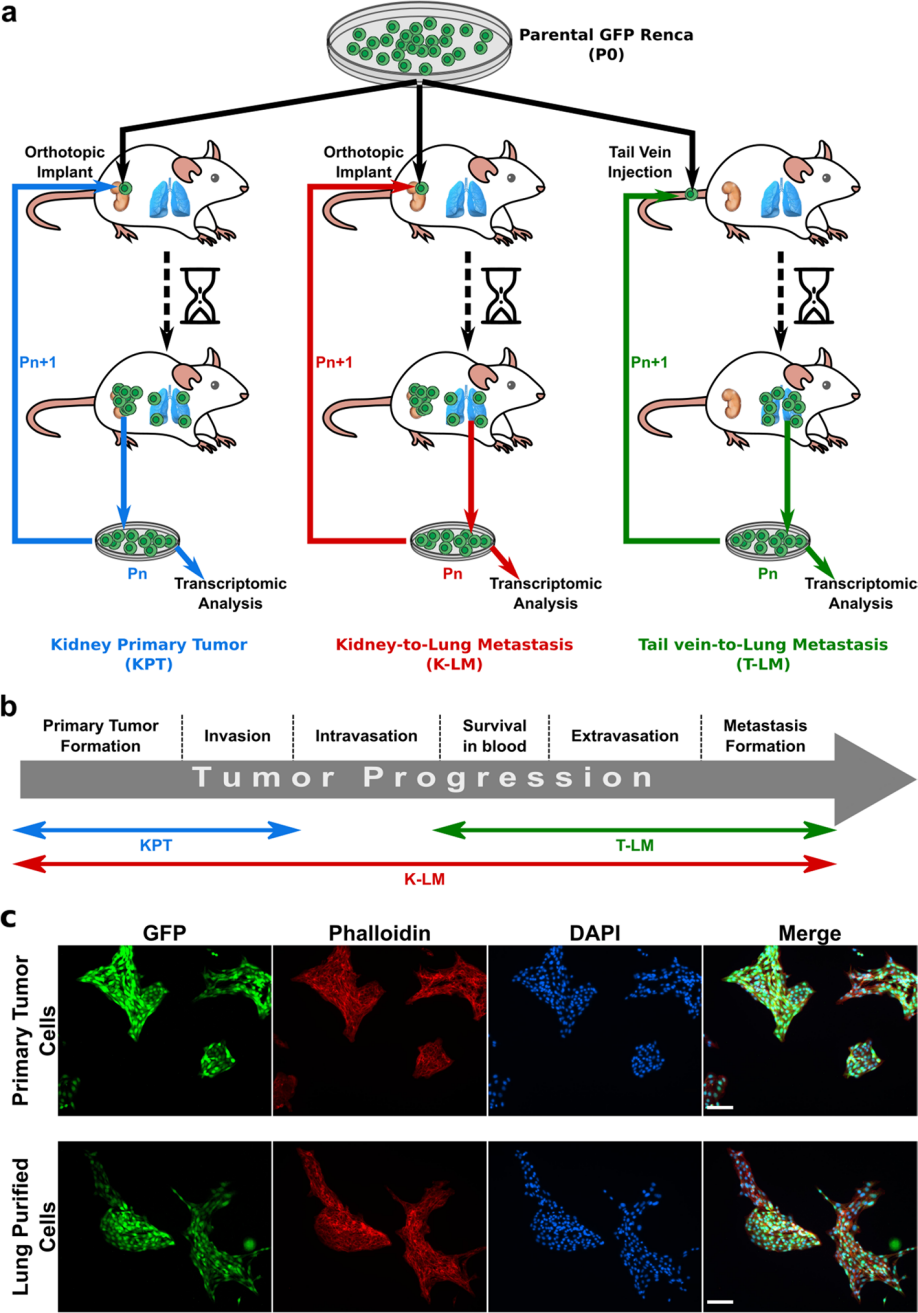
Design and development of the experimental model. **a** Three different GFP-RENCA implantation-extraction modalities were used to develop increasingly aggressive cancer cell lines: orthotopic implantation and extraction from primary tumors generated Kidney Primary Tumor cell lines (KPT); orthotopic implantation and extraction from lung metastases generated Kidney-to-Lung-Metastases cell lines (K-LM); injection into tail vein and extraction from lung metastases generated Tail-to-Lung-Metastases cell lines (T-LM). **b** Scheme of different tumor progression phases whose biological mechanisms are expected to be revealed by the generated cancer cell lines. **c** Immunofluorescence analysis of tumor- or lung metastases-explanted GFP-RENCA showing the absence of GFP-negative stromal cells after 14 days in culture. All cells were counter-stained with both Alexa Flour 568 phalloidin and DAPI. Scale bars, 100 μm. Pn, number of cell passage in vivo

To identify the molecular mechanisms responsible for the development of primary and metastatic tumors in RCC, we generated mouse renal carcinoma (RENCA) cell lines of progressively enhanced aggressiveness and metastatic potential, and analyzed the transcriptomic profiles. To this aim, we firstly generated RENCA cells stably expressing GFP, for cancer cells identification, and serially passaged the generated Green Fluorescent Protein RENCA (GFP-RENCA) cells in female BALB/c immunocompetent mice, based on the seminal work of Fidler [11]. For the dissection and study of each step involved in tumor progression, we used three different GFP-RENCA injection-explant modalities, coupled with RNA extraction from the generated cell lines for gene expression analysis. The three implantation/injection modalities are shown in Fig 1a and described as follows: (i) orthotopic implantation under the renal capsule; cancer cells were then explanted from formed tumors, and purified for subsequent re-implantation into a kidney of another mouse. (ii) intravenous injection into the tail vein, in the absence of a primary tumor in the kidney; here, cancer cells were explanted from the metastases formed in the lungs. (iii) orthotopic implantation under the renal capsule and GFP-RENCA collection from metastatic sites in the lungs; subsequently, cancer cells were re-implanted under the kidney capsule for the following passage(s). By this experimental strategy, we generated 67 different cell lines that can be grouped in three main categories, each one describing the different aspects of cancer progression, from primary tumor growth to metastasis formation (Fig. 1b): 1- Kidney Primary Tumor (KPT) cell lines, which were expected to reveal mechanisms relevant to primary tumor growth and invasion; 2- Tail-to-Lung Metastases (T-LM) cell lines, which described the key aspects of metastasis formation (i.e. survival in the bloodstream, evasion of immune response, colonization and growth in distant secondary organ, such as lungs); 3- Kidney-to-Lung metastases (K-LM) cell lines, that recapitulated the whole process of tumor progression, from primary tumor growth to metastasis formation.

To note, each cell line was sequentially passaged in vivo for 6 cycles, using multiple mice per injection mode and per passage. To purify GFP-RENCA cells from primary tumors and lung metastases at each passage, tissues were digested and cells were harvested and maintained in culture for 10–15 days before the next implantation (see Methods section). This window of time allowed non-cancer cell to die in order to reach a cancer cell purity equivalent to parental in vitro cultured GFP-RENCA cells (Fig. 1c, Supplementary Fig. S1) for the subsequent implantation or injection.

### Late passage P6 cell lines showed enhanced aggressiveness and metastatic potentials compared to earlier passage P1 cells

**Fig. 2:**
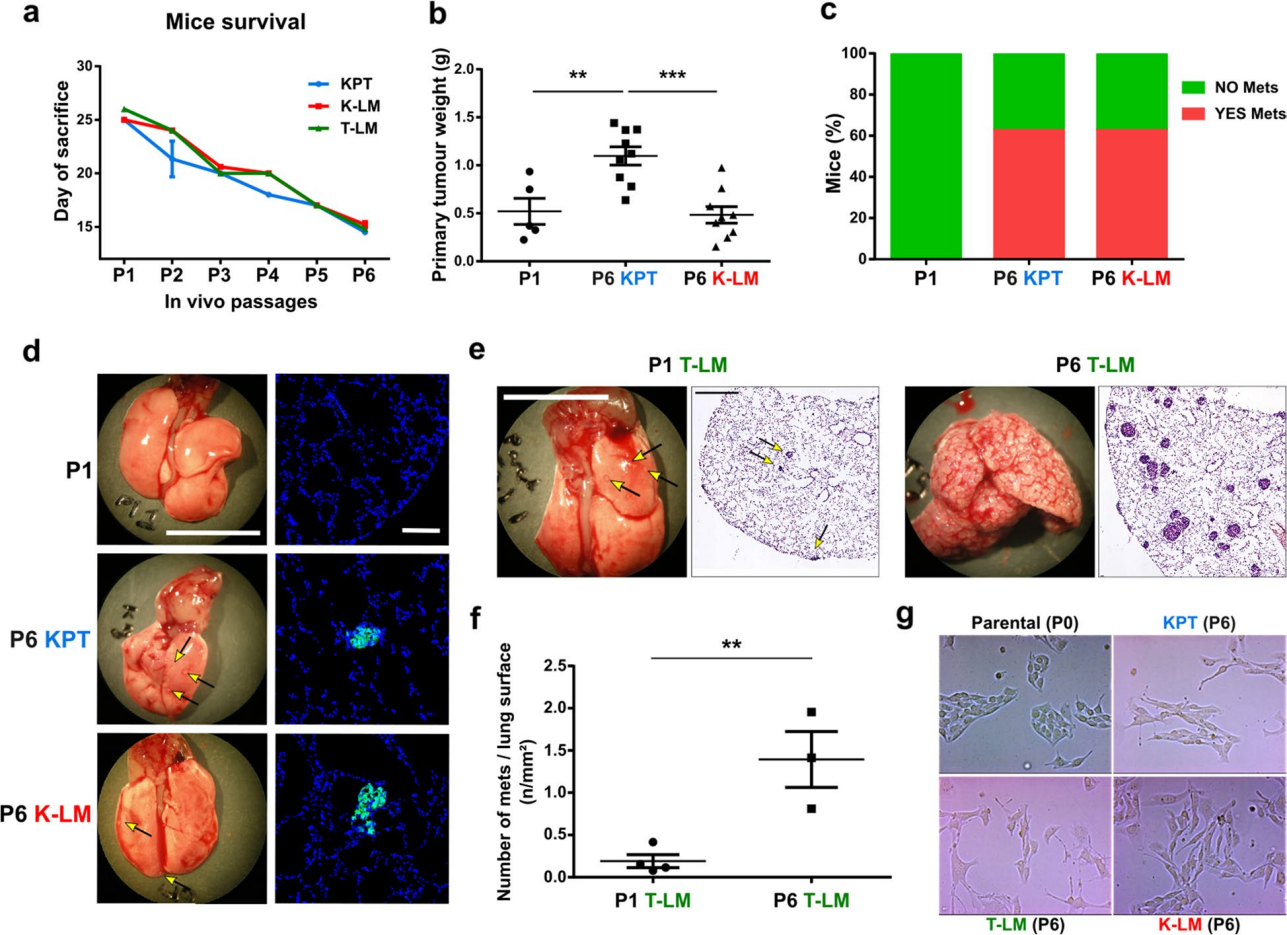
Enhanced cancer cell aggressiveness and metastatic potential of P6 cell lines. **a** Graph shows reduced mice survival, represented as time between day of cell implantation and day of sacrifice for reaching ethical endpoint(s), during cycles of GFP-RENCA implantation-extraction. **b** Dots graph shows the weight of primary tumors generated by P6 KPT or K-LM cells, compared to P1, 15 days after orthotopic implantation. **c** Bars graph showing the percentage of mice presenting, at the moment of sacrifice, visible metastases in the lungs, upon injection of late passaged GFP-RENCA (either P6 KPT or P6 K-LM) compared to orthotopically implanted P1 cell lines. **d** On the left (scale bar = 1 cm), representative pictures of visible lung metastases deriving from primary tumors generated by P1 or late passaged P6 KPT and K-LM cell lines. On the right (scale bar = 100 μm), representative immunofluorescent images showing GFP-positive lung metastases. **e** Representative pictures of lungs at the moment of sacrifice (scale bar, 1 cm) and hematoxylin-eosin staining (scale bar, 1 mm) of lungs bearing metastases formed after 15 days from P1 versus P6 cells that were injected into tail vein (i.e. T-LM cell lines). **f** Number of metastases, per lung surface, generated by T-LM P6 cells, compared to P1, in tail vein injected mice. **g** Light microscopy images showing difference in mode of growth of P0 versus P6 cell lines grown in 2D-culture. Arrows indicate the presence of metastases on lung surface. Data are represented as mean ± SEM

During the generation of the cell lines, we observed a passage-dependent reduction in mice survival time (from 26 to 15 days), suggesting that the cells became increasingly aggressive after each in vivo implantation/extraction cycle (Fig. 2a). After 15 days from orthotopic implantation, P6 KPT cells formed larger primary tumors compared to GFP-RENCA that underwent only one cycle of injection (i.e. P1 cells). However, P6 K-LM cell lines generated tumors that were of comparable weight with the ones formed by P1 cells (Fig. 2b). Such a difference in tumor growth could be explained by an increase in the proliferation rate of P6 KPT cells, as shown by immunofluorescence analysis of the proliferative marker Ki67 in these samples (Supplementary Fig. S2a, b). Concomitantly with primary tumor growth, we also compared the metastatic potential of P6 cell lines to P1 cells. In particular, we observed that P6 cells enhanced the formation of lung metastases compared to P1 cells, after 15 days from either orthotopic implantation or tail vein injection (Fig. 2c-f). Furthermore, P6 cells changed their mode of growth in 2D-culture. In fact, RENCA cells normally form compact colonies. However, P6 cells were less adherent to each other, compared to parental non-implanted P0 cells, and unable to grow in clusters (Fig. 2g). Such a phenotype, in addition to increased in vivo metastatic potential, suggested that P6 cells acquired an enhanced migratory ability, as further demonstrated by a Boyden chamber assay (Supplementary Fig. S2c). Furthermore, gene expression analysis of P6 of the 3 different groups revealed changes reminiscent for an EMT phenotype (Supplementary Fig. S2d). In addition, we investigated some cancer stem cell markers and we demonstrated an increase in Cd44, Nt5e and Aldh1a1 (Supplementary Fig. S2e).

### Global functional genomic analysis of amplified mouse cell lines

In order to determine whether the phenotypic differences are due to genomic or epigenetic alterations, we first performed low-coverage whole-genome sequencing on P0 and P6 cells lines to assess whether copy number variability could possibly underlie the change in phenotype (Supplementary Fig. S3). We failed, however, to observe significant differences in copy numbers between parental and passaged samples, both at the level of the number of breakpoints detected (45 for parental versus 41.75 ± 6.32 for passaged lines) and the percentage of the genome with a copy number different than 2 (19% for parental versus 18% ± 1% for passaged lines) (see Figure description for details).

**Fig. 3:**
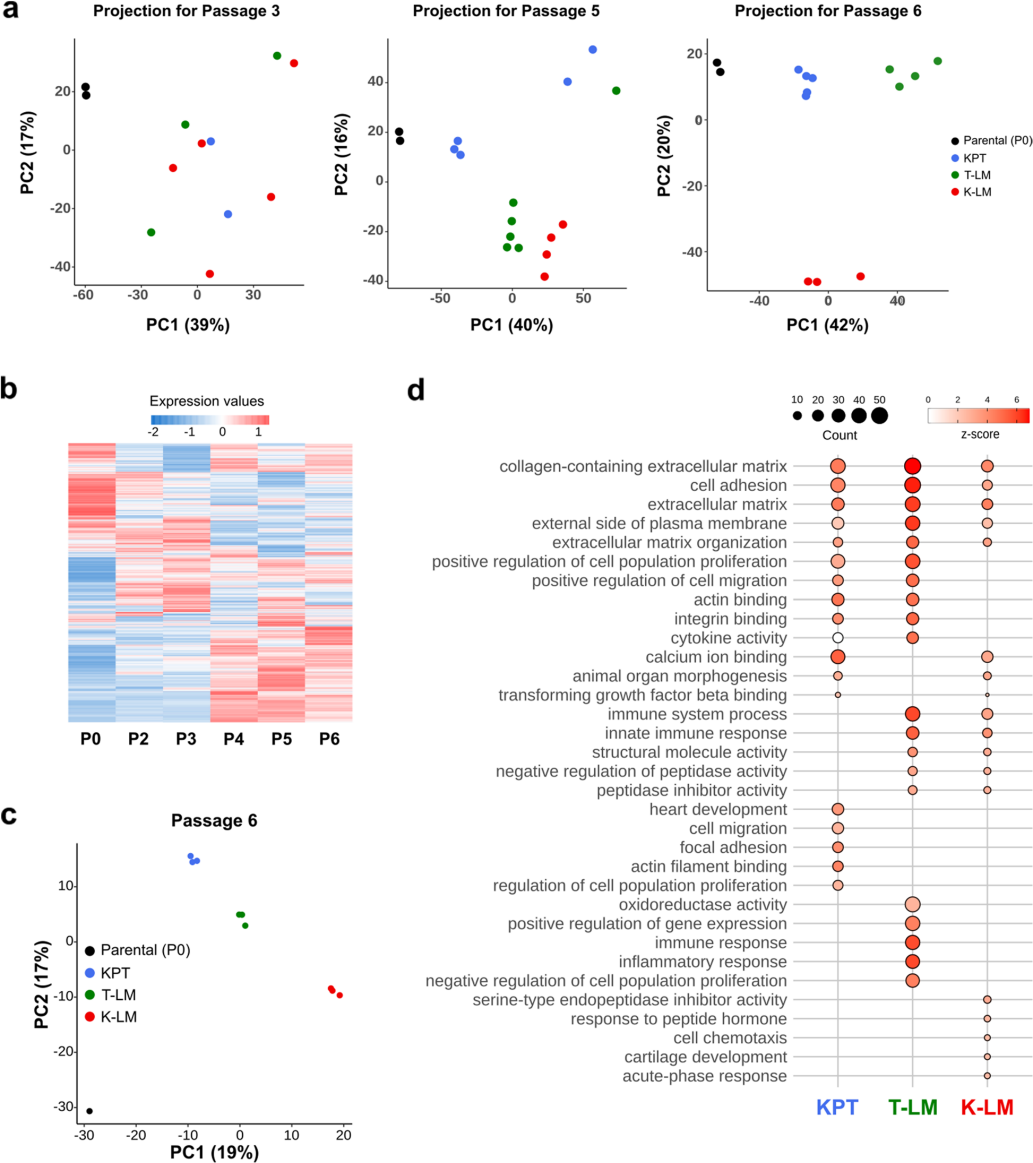
Analysis of transcription datasets. **a** Projection of samples onto principal component 1 and 2 (PC1 and PC2) for the P0-P3, P0-P5 and P0-P6 principal component analysis (PCA) on transcriptomics values. Efficient samples clustering occurred after 6 cycles of in vivo passaging. **b** Heatmap for PC1- and PC2-associated gene expression from the P0-P6 PCA displaying a progressive pattern. **c** Projection of samples onto PC1 and PC2 for the P0-P6 PCA on methylation frequencies of all CGs from the whole genome methylation sequencing. **d** Top 5 significantly enriched GO terms (adjusted *p*-value ≤0.05) for differentially expressed genes in each group, between passages P3 and P6. Top 5 of each group and combined group are depicted. The z-score indicates how genes are differentially expressed according to GO terms: a positive z-score indicates that the number of up-regulated genes is higher than the number of down-regulated genes in the respective GO term. Counts represent the number of genes which are differentially expressed

As the differences cannot be explained by genomic alterations we focused on transcriptomic and methylome analysis. We performed RNA extraction of GFP-RENCA cells isolated after each passage for transcriptomic analysis. We used Principal Component Analysis (PCA) to summarize the information contained in our data sets for cell passages P3, P5 and P6. This analysis revealed that, after each passage, clustering became more evident and cluster segregation specific for the respective implantation/injection mode. Thus, at latest passage P6, KPT, T-LM and K-LM cell lines clustered into three distinct groups (Fig. 3a). We next selected all the genes that were major contributors to the Principal Component 1 (PC1) and 2 (PC2) and performed a heatmap of the transcriptomic profiles. The P1 cell lines were excluded from the analysis because of insufficient number of replicates. This analysis revealed a gradual change in gene expression along with cell passages (Fig. 3b). The PCA for the methylome data obtained by full methylome sequencing of P0 and P6 cells, showed similar clustering in 4 groups corresponding to KPT, T-LM, K-LM and parental P0 cell lines (Fig. 3c).

Gene Ontology (GO) enrichment analysis between P3 and P6 cell lines (see Materials and Methods for details) showed several highly enriched categories for each group (Fig. 3d). These include processes that are in common for all three groups, for 2 groups or specific of each group. Common processes such collagen-containing ECM, cell adhesion, or extracellular matrix organization are required for cancer cells to evolve during the different steps of tumor progression. Others were more specific for one or two group(s) only, and are in favor of role in either primary tumor growth, metastatic spread or survival in the blood stream during the dissemination process or a combination of two of these processes. These include for instance categories such as regulation of cell population proliferation, positive regulation of cell migration, actin-binding, integrin binding, cytokine activity, Transforming Growth Factor-b (TGF-β) binding and immune system processes. This further indicates that the three groups of cell lines evolved differently up to the passage P6 by acquiring different regulatory modules.

Inactivating mutation of the von Hippel-Lindau (VHL) gene is a hallmark of renal carcinoma cells which is detectable in the majority of RCC patients. Despite the fact that our model of RCC was generated using a wild-type VHL cell line (i.e. RENCA), we observed that T-LM and K-LM late passage P6 cell lines displayed main characteristics of mutated VHL RCC cells (i.e. enhanced metastatic potential and EMT). We took advantage of a previously published study, where a set of upregulated gene with clinical impact has been identified in VHL-knockout RENCA cells [12].

To assess the degree of VHL cascade activation in our cell lines, we compared the expression of this VHL knockout related genes to our transcriptomic dataset. The heatmap of T-LM and K-LM late passages cells (including P4-P5-P6) revealed clustering and increased expression of the VHL knockout related genes when compared to early passaged cells (Supplementary Fig. S4a). Particularly, these included four Hif1α target genes (i.e. POSTN, TNFSF13B, PPEF1 and SAMSN1) that were significantly up-regulated in VHL-KO RENCA, and are of poor prognosis for RCC patients (Supplementary Fig. S4b). Only the KPT cell lines did not show evident clustering with the VHL-KO RENCA gene signature. To elucidate whether such transcript changes could indeed depend on differences in Vhl, Hif1a or Hif2a levels, we analyzed by qPCR the expression of these three genes using RNA extracted from P0 and P6 cells. As shown in Supplementary Fig. S4c, in late passage P6 cells, Vhl gene was downregulated for all groups and Hif2a was up-regulated mainly in K-LM and T-LM groups, while Hif1a expression was slightly decreased in KPT cells.

### Clinically relevant signatures and biomarker discovery

We next investigated whether transcriptional signatures derived from the analysis of differentially expressed genes in the KPT, T-LM and K-LM- groups could predict clinical outcome of patients. To this aim we used the Clear Cell Renal Cell Carcinoma dataset (KIRC) from The Cancer Genome Atlas (TCGA).

**Fig. 4:**
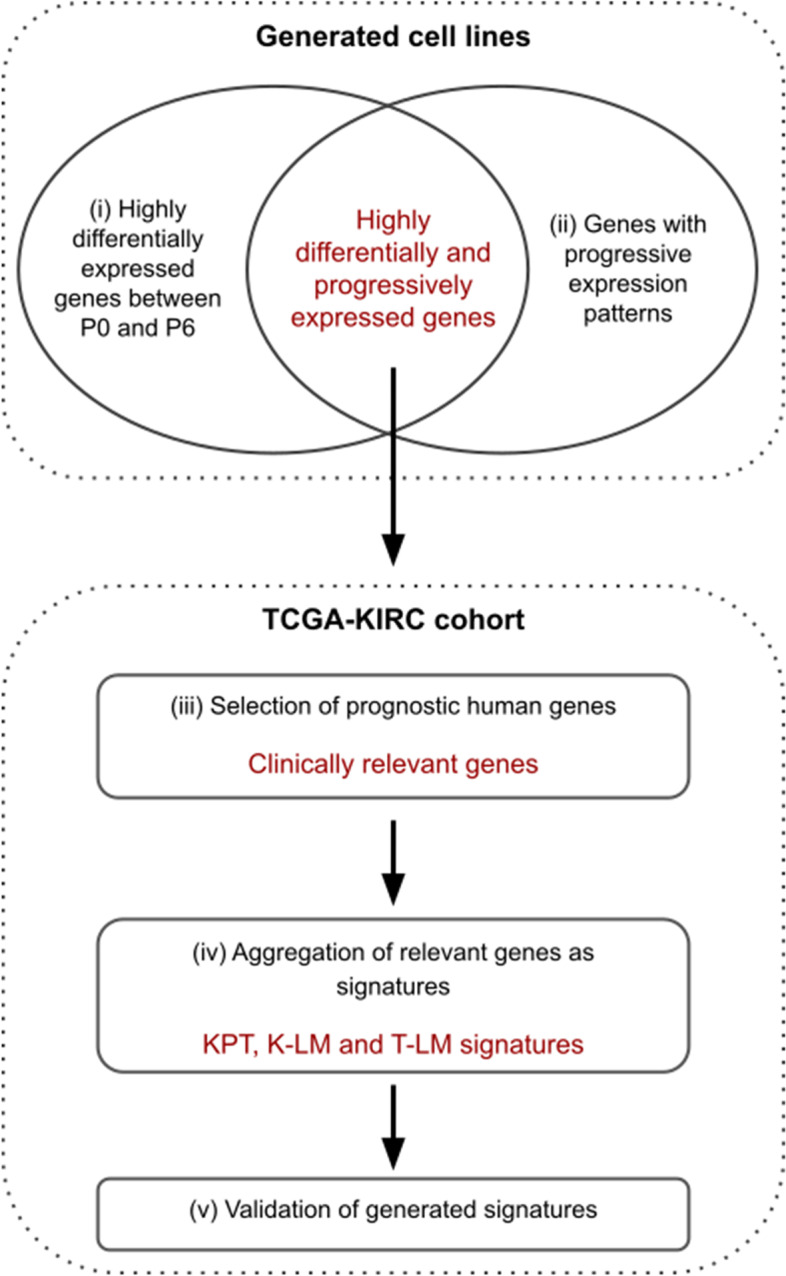
Strategy used for RCC biomarkers discovery and validation. The Fig. depicts the general strategy used to determine clinically relevant signatures from each generated cell line (i.e. KPT, K-LM and T-LM). For detailed description see Materials and Methods section

The general strategy is outlined in Fig. 4. To select biomarkers from our mouse model, we compared genes that changed their expression between the parental P0 and P6 cell line and exhibited a progressive expression pattern (KPT, T-LM, K-LM; Supplementary Table S1).

To determine the list of clinically relevant genes, we next investigated the predictive value of the 3 gene lists using TCGA (KIRC cohort). For each gene, we fitted a Cox-proportional hazard regression model based on overall survival (OS) or disease-free survival (DFS). A gene was conserved if its false-discovery rate (FDR) adjusted *p*-value of its log-rank test was lower than 0.01 and if the hazard ratio (HR) was consistent with the differential expression (Supplementary Table S2).

To determine the specific signatures, we combined genes from the clinically relevant biomarker gene list. In order to validate our signature, we computed an empirical p-value by testing our signatures against 1000 random signatures of equivalent size. Furthermore, we performed multivariate Cox regression analysis of our signatures. The results for the 3 subgroups are depicted including the OS and DFS for M0 patients (Table 1, Supplementary Fig. S5, S6, S7). After adjusting for clinical variables (TNM stage and Fuhrman grade), the K-LM signature had the best hazard ratio and remained an independent prognostic factor for predicting both OS and DFS.

**Table 1:** KPT, K-LM and T-LM signatures

Signature	Genes	OS					DFS				
		HR (95% CI)	HR-logRank (95% CI)	***p*** value 2 arms	p value 3 arms	Empirical P value	HR (95% CI)	HR-logRank (95% CI)	p value 2 arms	p value 3 arms	Empirical P value
**KPT (*N* = 14)**	ADAM8, ARHGAP33, BTG3, COL6A1, CYBA, DNAJC12, DYRK4, FKBP10, IL34, MTMR7, PADI3, PLAU, RCN3, TPRG1	5.081 (3.313–7.791)	4.968 (3.495–7.06)	1.20E-16	1.17E-17	0	3.694 (2.049–6.658)	3.605 (2.168–5.992)	3.40E-06	1.85E-07	0.004
**T-LM (*N*** **= 24)**	ARHGAP33, BTG3, C4orf48, CKAP4, CRABP2, CYP3A4, DNAJC12, DYRK4, EREG, GFPT2, HIST1H1E, KCTD17, KDELR3, MMP14, NCAM1, NME4, PIGZ, PLAU, PLOD2, RGS19, SERPINA3, TBX4, TMEM45A, TPRG1	4.734 (3.086–7.262)	4.576 (3.215–6.512)	1.00E-14	1.15E-15	0.001	4.293 (2.314–7.964)	4.096 (2.456–6.833)	1.03E-06	1.95E-08	0.003
**K-LM (*N*** **= 16)**	BCL3, CFB, COL6A1, CYP3A4, IQSEC3, KCTD17, KRT19, LOXL2, LRG1, PCBP3, SAA1, SAA2, SERPINA3, SOCS3, UCK2, WT1	4.102 (2.666–6.313)	4.002 (2.786–5.749)	5.91E-12	3.94E-11	0.006	4.752 (2.381–9.484)	4.59 (2.664–7.91)	1.82E-06	6.96E-06	0.003

We next compared our signature in the data set reporting treatment of RCC by targeted therapy which included immunotherapy [13]. All three signatures (KPT, K-LM, T-LM) were predictive for OS when assessed in the cohort treated with immunotherapy (Supplementary Fig. S8).

The identification of signatures predictive of patient outcome also validates our experimental approach and shows that the strategy of generation of increasingly specialized mouse cell lines revealed novel genes and signatures with relevance to human RCC.

### Validation of serum amyloid A2 (SAA2) and complement factor-B (CFB) and elaboration of a computational model

#### Validation of SAA2 and CFB

To identify novel potential prognostic or diagnostic markers or therapeutic targets, we focused on the K-LM signature, because it is related to metastatic spread and had the highest Hazard Ratio (HR). Among the genes found in this signature, we focused on SAA2 and CFB because they represent soluble markers that can be analyzed in the blood. SAA2 is an acute phase protein related to SAA1, which was previously linked to metastasis [14], while CFB, is part of alternate pathway of the complement system. Both have never been described in RCC.

**Fig. 5:**
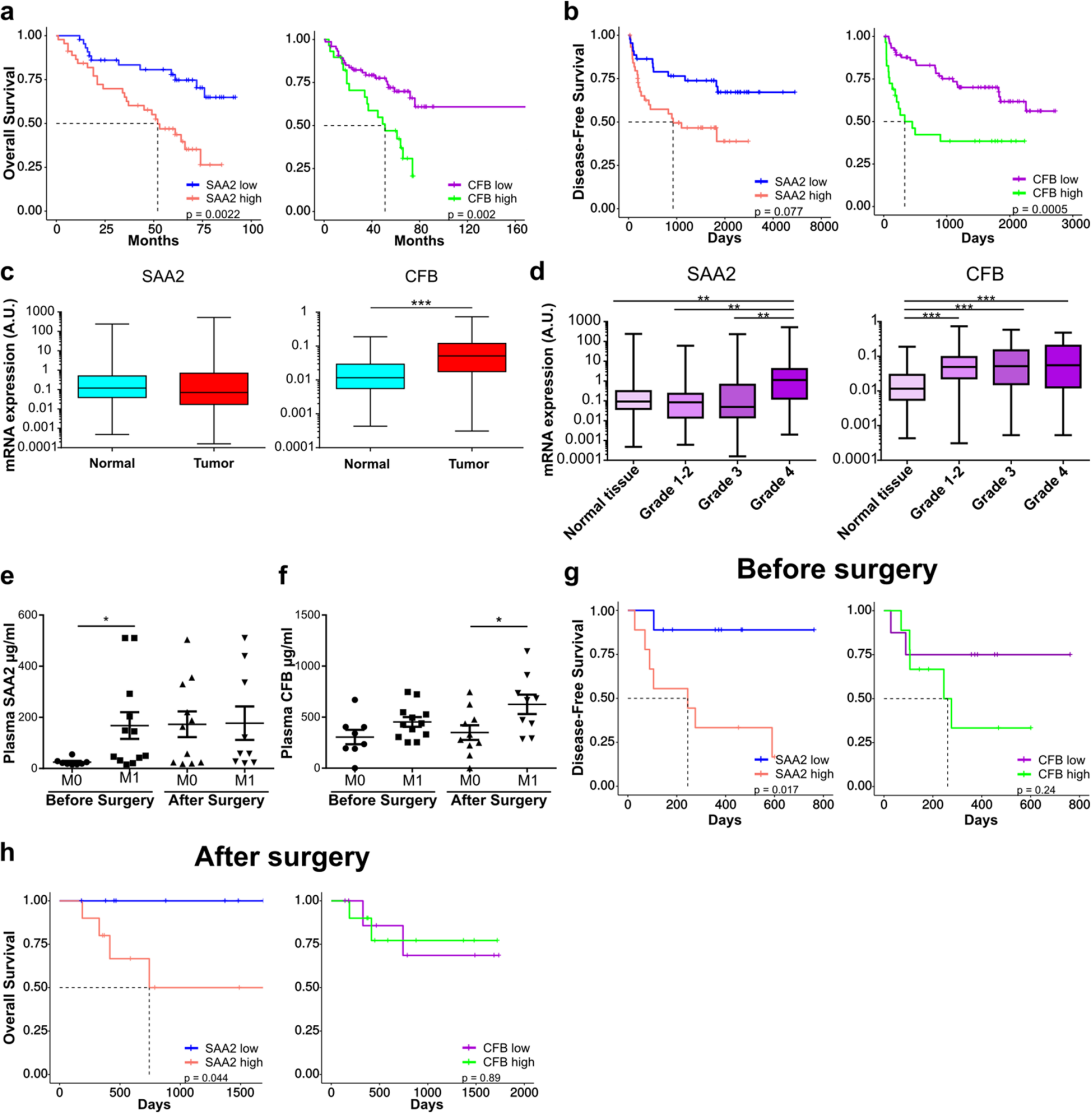
Clinical relevance of SAA2 and CFB expression in the UroCCR cohort. **a** Overall Survival (OS) of patients stratified according to SAA2 (HR(log-rank) = 2.901 (1.526–5.517)) or CFB gene expression (HR(log-rank) = 2.556 (1.24–5.267); *n* = 89). **b** Disease-Free Survival (DFS) of patients stratified according to SAA2 (HR(log-rank) = 2.342 (1.211–4.529)) or CFB gene expression (HR(log-rank) = 2.846 (1.323–6.123); *n* = 104). **c** qPCR analysis of tissues deriving from primary tumors or adjacent normal tissues. **d** Gene expression of SAA2 and CFB in patient’s tumor tissue stratified according to low Fuhrman grade (1–2), grade 3 and grade 4, compared to adjacent normal tissues. **e** and **f** ELISA experiment showing plasma levels of SAA2 and CFB, in non-metastatic (M0) and metastatic (M1) patients at the moment of diagnosis, before and after surgery, respectively. **g** Disease-Free Survival (DFS) of patients stratified according to SAA2 (HR(log-rank) = 8.191 (2.04–32.891); Low, *n* = 9; High, n = 9) or CFB (HR(log-rank) = 2.545 (0.578–11.201); Low, n = 8; High, n = 9) plasma levels before surgery (i.e. primary tumor resection). **h** Overall Survival (OS) of patients stratified according to SAA2 (HR(log-rank) = Inf, not calculable) or CFB (HR(log-rank) = 0.875 (0.123–6.237)) plasma levels after surgery (i.e. primary tumor resection). Low, n = 9; High, n = 10

Validation of both markers has been carried out in the nation-wide renal cell cancer tumor collection (UroCCR cohort) which confirmed their prognostic value on OS and DFS when using mRNA levels (Fig. 5a, b). mRNA expression of SAA2 was similar in healthy and tumor samples, but CFB was significantly more expressed in tumor samples. (Fig. 5c). When expression was analyzed according to the Fuhrman grade, SAA2 showed significant higher levels in grade 4 when compared to grade 2 or 3, but for CFB no difference was seen (Fig. 5d). We next measured SAA2 and CFB levels in plasma samples from patients with (M1) or without metastases (M0), collected before or after surgical resection of the primary tumor. M1 patients had higher plasma levels of SAA2 before surgery and equivalent levels after surgery, whereas the levels of CFB were not increased before surgery but significantly higher after surgery (Fig. 5e, f). When patients were divided into two groups of equivalent size, the group with higher SAA2 levels had a shorter survival when levels were measured before (DFS) and after surgery (OS) (Fig. 5g, h). For CFB, DFS or OS were not significantly changed, albeit a tendency for DFS was recorded.

**Fig. 6:**
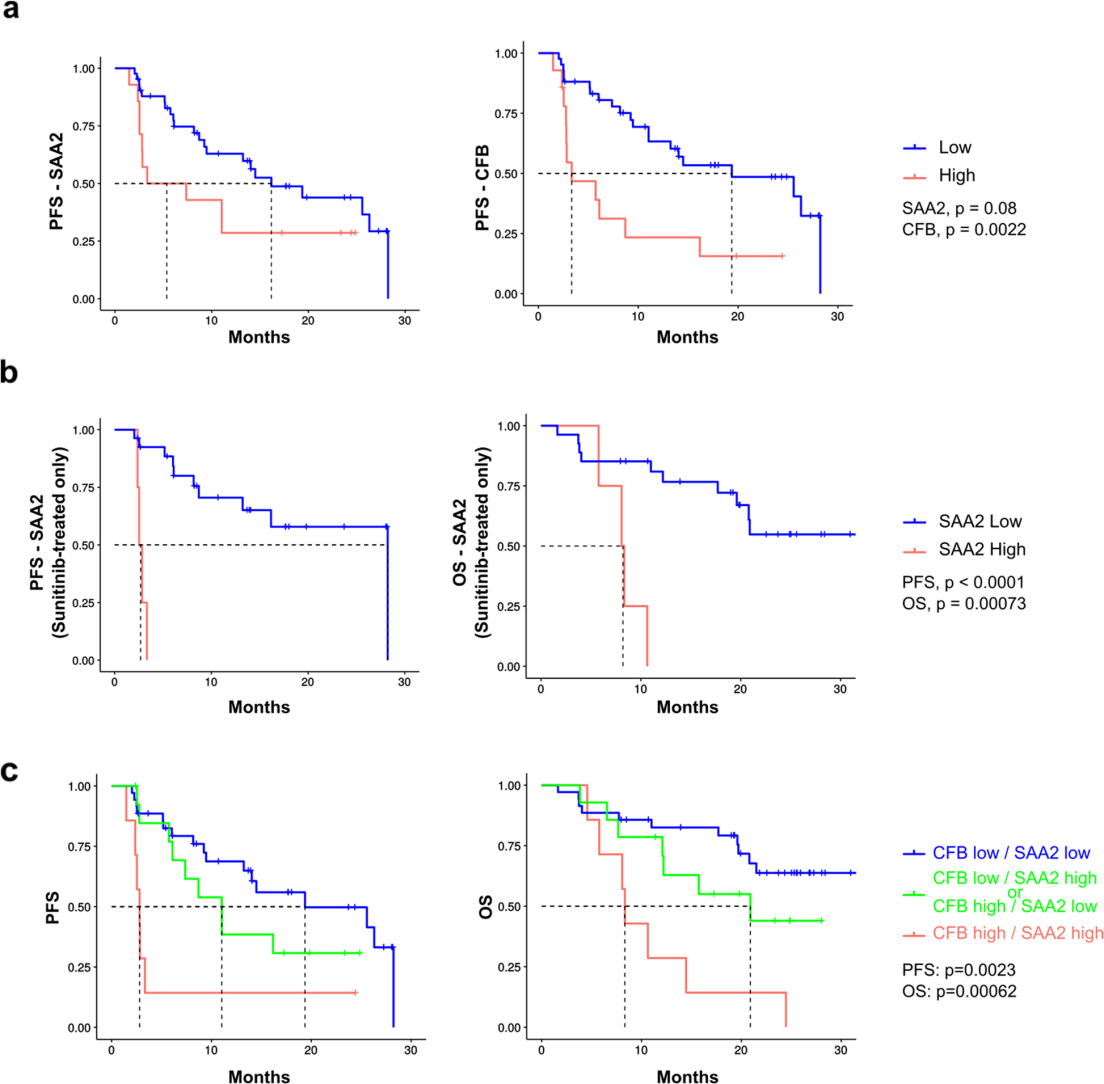
Clinical relevance of SAA2 and CFB expression in patients treated with antiangiogenic therapy (SUVEGIL-TORAVA cohorts). **a** Association between plasma SAA2 or CFB levels at diagnosis and Progression-free survival (PFS) in patients after sunitinib or bevacizumab treatment (plasma level at diagnosis less or greater than a 2nd quartile cut-off for: SAA2 (196.83 μg/ml; HR(log-rank) = 2.309); CFB (266.03 μg/ml; HR(log-rank) = 2.078). Low, *n* = 45; High, *n* = 14 **b** Progression-free and overall survival (left and right, respectively) of patients, stratified according to plasma levels of SAA2 at diagnosis, after treatment with sunitinib (plasma level at diagnosis less or greater than a cut-off for SAA2 (196.83 μg/ml)(OS: HR(log-rank) = 6.922; Low, *n* = 30; High, *n* = 4. PFS: HR(log-rank) = 8.035. Low, *n* = 27; High, n = 4). **c** Three subgroups were identified *i)* CFB low and SAA2 low, *ii)* CFB low and SAA2 high or CFB high and SAA2 low, *iii)* CFB high and SAA2 high. Low-low vs high-high: PFS HR(log-rank) = 4.324); OS HR(log-rank) = 3.373. PFS (left graph; Low, *n* = 38; Low/High or High/Low, n = 14; High, *n* = 7) and OS (right graph; Low, *n* = 35; Low/High or High/Low, n = 14; High, n = 7) of patients treated with either Sunitinib or bevacizumab and stratified according to SAA2 and CFB plasma levels

We next used plasma samples from metastatic patients before receiving a first cycle of sunitinib or bevacizumab (SUVEGIL, NCT00943839 and TORAVA, NCT00619268 clinical trials). The analyses were performed at the third quartile. Patients treated with anti-angiogenic drugs (sunitinib and bevacizumab) and stratified according to low and high SAA2 CFB levels, had a spectacular better PFS when belonging to the SAA2 / CFB low group (cut-off: SAA2 272.49 μg/ml; CFB 311.96 μg/ml). When patients treated with sunitinib alone were analyzed, the PFS and OS was significantly shorter in the high expression group (Fig. 6a, b).

When SAA2 and CFB were combined, we can subdivide the cohort into 3 different groups and rank PFS and OS accordingly: CFB low/SAA2 low (PFS 13.23 months, OS: 20.8 months), CFB low/SAA2 high or CFB high/SAA2 low (PFS 9.87 months, OS 16.52 months), and CFB high/SAA2 (PFS 2.8 months, OS 8.33 months) (Fig. 6c).

Thus, the combined analysis of these two markers is a powerful predictor of patient outcome following anti-angiogenic treatment with sunitinib or bevacizumab.

We also investigated CFB and SAA2 in the cohort of the published dataset treated with immunotherapy (Supplementary Fig. S9). For immunotherapy with anti-PD1, CFB and SSA2 alone were not significant predictor. However, the combination of CFB and SAA2 was found to be predictive for OS.

#### Computational Modelling

**Fig. 7:**
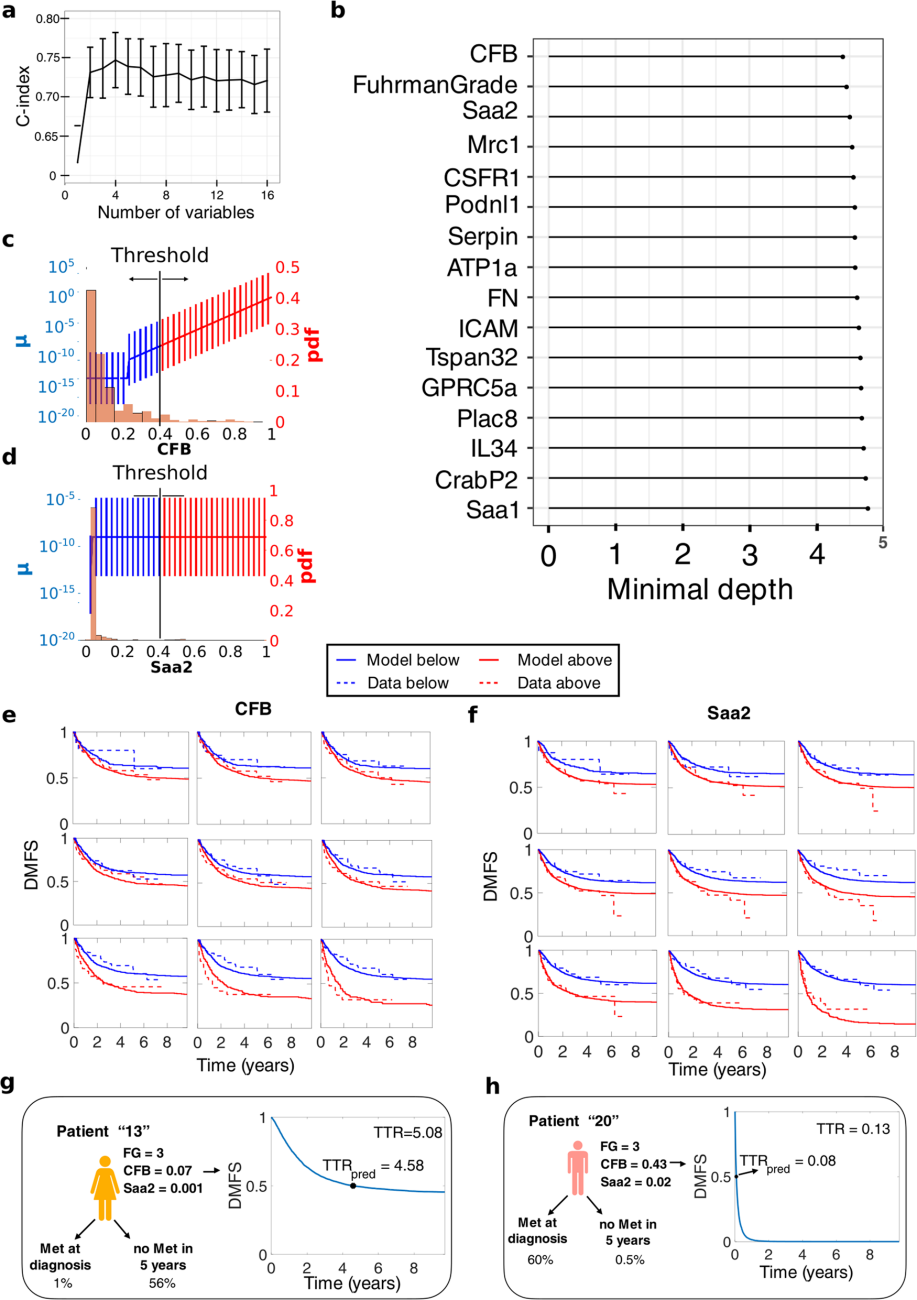
Computational analysis for prediction of time to relapse (TTR).**a** Cross-validated Harrel’s C-index using random survival forest models. The variables are selected by importance using minimal depth. **b** Minimal depth ranking of covariates. **c-d** Effect of the covariate in metastasis dissemination according to the value of the covariate. The histogram corresponds to the covariate data and the vertical bars are the corresponding values of the metastasis dissemination parameter (μ) distribution according to the value of the covariate. (See Ref. A8 for more details). **c** CFB, specific parameter values are b = 1.04 (Relative Standard Error (RSE) = 14.90%), c = 0.22 (RSE = 24.26%) and dif = − 0.67 (RSE = 11.44%) **d** Saa2, specific parameter values are b = 0.32 (RSE = 32.82%), c = (RSE = 39.47%) and dif = − 0.89 (RSE = 61.98%). **e** ﻿Goodness-of-fit for the model with the effect of CFB and the data at different thresholds. **f** Goodness-of-fit for the model with the effect of SAA2 and the data at different thresholds. **g-h** Individual predictions of two patients, with probabilities to have metastasis at diagnosis or not to have metastasis after 5 years. The plot corresponds to the predicted DMFS curve for the individual patients, which allows to calculate the predicted TTR

Machine learning and mathematical modeling analysis further confirmed the importance of these covariates. CFB and SAA2, together with Fuhrman Grade, were the three covariates with the highest impact on distant metastasis-free survival (DMFS) in random survival forest analysis, see Fig. 7 a, b. We then analyzed those covariates with a mathematical model of tumor growth and metastasis dissemination [15–17]. Using the mathematical model of TTR and the analysis briefly described in Methods and fully described in Álvarez-Arenas et al. [18], we concluded that the impact of CFB and SAA2 on TTR was more likely through a direct effect in metastasis dissemination rather than in tumor growth. In addition, the mathematical model suggested that for higher values of CFB, the effect on metastatic dissemination (parameter μ) increases linearly, while for SAA2 its effect is stratified into two groups (see Fig. 7c, d). The mathematical model accurately described dichotomized Kaplan-Meier DMFS curves for CFB and SAA2, see Fig. 7e, f respectively. We then combined these two covariates with Fuhrman grade into a single model to make individual predictions. For a given patient with specific values of CFB, SAA2, and Fuhrman grade, we simulated the TTR of 10,000 in-silico replicates. Each replicate accounted for uncertainty in the volume of the primary tumor at diagnosis and the remaining unexplained inter-individual variability. With the virtual values of TTR, it was possible to compute personalized distant metastasis free survival (DMFS) curves for each patient. Moreover, other information such as the probability to have metastasis at diagnosis or to be metastasis-free after 5 years could also be computed. From personalized DMFS curves, we then took the median of the 10,000 TTRs and defined it as the predicted TTR. With the predicted TTR for all the patients in the dataset, together with the real TTR, we calculated the C-Index of the models with different combination of these three covariates. The best C-Index was achieved with the model combining Fuhrman grade and CFB (C-Index = 0.7273), followed by the three covariates together (C-Index = 0.7163) and Fuhrman grade alone (C-Index = 0.6948). To compare, we also computed the C-Index using multivariable Cox regression. In all cases, the C-Index was similar to the mathematical model, see Table 2.

**Table 2:** C-index comparison between mechanistic and cox-based model considering different covariates. Confidence intervals were calculated bootstrapping the data 100 times and using the percentile method

C-Index	Mechanistic Model	Cox-based model
FG	0.6948 (0.5664–0.7551)	0.7143 (0.6349–0.7808)
FG/CFB	0.7273 (0.6403–0.7998)	0.7434 (0.6781–0.8318)
FG/CFB/Saa2	0.7163 (0.6541–0.7802)	0.7437 (0.6918–0.8159)

## Discussion

The incidence and prevalence of RCC are rising with a high mortality for metastatic disease. Different drugs are available for RCC treatment, aiming to counteract its high angiogenic and immunogenic environment [19]. However, the limitation associated with these drugs includes treatment resistance leading to failure of RCC eradication. The pathophysiology of RCC is still far from understood, and treatment of RCC is hampered by a lack of validated molecular biomarkers. The elucidation of key mechanisms in RCC progression and the identification of novel biomarkers of RCC can open up novel therapeutic strategies targeting different aspects of RCC biology, including chemo-resistance.

We have generated a mouse model designed to recapitulate different stages of RCC progression. The RENCA model has been widely used in many preclinical RCC studies and has led to meaningful results [20–23]. This cell model could represent a weak point of our experimental approach, as they bear wild-type VHL gene status. In fact, the majority of human RCC tumors are inactivated for VHL. However, when comparing the transcriptome profiles of our cell lines with VHL-KO RENCA cells, generated in a previous study by Schokrpur et al. [12], we observed that late passages K-LM and T-LM cells exhibit an increase in the expression of an identical set of genes observed in VHL-KO RENCA cells. These data indicate that our late passages K-LM and T-LM are suitable for describing the molecular changes occurring in VHL-mutated RCC.

Alternatively, to study the biological processes driving RCC progression in patients, other human RCC cell lines could be used (either VHL wildtype cells (ACHN, Caki2) or VHL mutated cells (RCC4, RCC10, 786-O, A498) [24]. However, the implantation of human cell lines or patient derived xenografts (PDX) requires longer time to develop, and, besides, uses immunodeficient mice. Since the immune system has a crucial role in tumor cell dissemination, metastatic formation and potential chemo-resistance, human cell lines or PDX models are not well suited since they focus mainly on primary tumor growth in an immuno-compromised host. Several genetically engineered mouse model (GEMMs) for RCC have also been developed [25] such as the MYC oncogene activation, Vhl-del, cyclin Cdkn2a-del, Ink4a/Arf-del model [26], the BRCA1 associated protein-1 (Bap1) and Polybromo 1 (Pbrm1) inactivation model [27]. Only the Vhl-del Ink4a/Arf-del model produced some metastasis, but only in the liver. However, no suitable GEMMs of RCC recapitulating with high fidelity the metastatic process, as our model does, exists to date [28–31].

To decipher the different steps of renal tumor development and dissemination, we generated mouse renal cancer cells of progressively enhanced aggressiveness and specialization. Using multiple implantation strategies, we analyzed different aspects of primary tumor growth and metastasis, and determined their transcriptomic profiles. This approach allowed monitoring of changes in gene expression during RCC development and progression, from primary tumor growth up to metastasis formation.

Renal cell carcinoma spreads mainly to the lung, and, at a lower rate, to other organs such as liver, bone, and brain [32]. The aim in our article was to specifically focus with our model on the primary metastasis site, the Lung. We have set-up two different metastasis models because they depict different situations as shown in Fig. 1 of the manuscript. The K-LM group reflects the full metastatic process including local invasion, intravasation, survival in the blood stream and extravasation and seeding into the lung tissue. The T-LM group only depicts the later stages of the process (survival in the blood stream, extravasation and seeding). We generated three distinct lists of differentially expressed genes, based on the implantation modality used. Genes were then selected based on their prognostic values in TCGA-KIRC cohort, and signatures were extracted and finally validated.

Although serial tumor cells passaging in mice is an established technique to generate more aggressive cell lines, we are the first to analyze the transcriptional changes of the different cell lines. RNA expression and methylome analysis demonstrated distinct clustering for the three different injection modes. DNA sequencing did not show clonal variations based on chromosomal variability, which indicates that the phenotypic changes were epigenetically regulated. Transcriptomic analysis led to the identification of specific gene signatures for each injection mode which were predictors of OS, DFS and PFS in ccRCC based on the TCGA-KIRC cohort and on our ccRCC cohorts. Importantly, the signatures, especially the K-LM signature, were stronger predictors than current predictors in clinical use such as Fuhrman grade or clinical stage. Our signature is different from the signature previously published such as ccrcc1–4 signatures [33], 16-gene assay [34] and ClearCode34 [35]. Ricketts et al. (29) reported a comprehensive molecular characterization using the TCGA database where they compared the three types of RCC (ccRCC, papillary RCC and chromophobe RCC). For ccRCC, the signatures were related to increased ribose metabolism pathway and to Th2 immune profile. However, our study is very different because starting from an animal model, we specifically focused our comparative translational analysis on the ccRCC subtype. Thus, the results cannot be compared, albeit their study also revealed immunology-related genes. Furthermore, a recent study reported tracking of ccRCC evolution at the genomic level and demonstrated that metastatic competence was afforded by chromosomal complexity with loss of 9p as a selective event for metastasis and patient survival [36]. However, our study did not reveal chromosomal alterations and, thus, we specifically focused on modifications in gene expression.

Our signatures were also found to be predictive when analyzed in an immunotherapy data set which could of use in stratifying patients undergoing targeted therapy.

For a more detailed investigation, we selected SAA2 and CFB, because they represent soluble and measurable proteins in the blood, that have never described in RCC. SAA2, an acute phase protein related to SAA1, was found to be a strong predictor of OS and DFS in the TCGA-KIRC database globally and also for the M0 and M1 subgroups in UroCCR cohort. In patients with the highest Fuhrman Grade, a significantly increased SAA2 expression was observed. Furthermore, analysis of plasma samples from patients with metastases before or after surgery showed higher plasma levels of SAA2 and worse OS. Analysis of samples from the clinical trials evidenced SAA2 as an excellent predictive biomarker especially in the sunitinib treated patient cohort. Previously, Vermaat et al. showed that SAA proteins were prognostic marker in RCC [37–39]. However, their study did not discriminate the prognostic significance of each SAA variants (SAA1–4). In addition, in their first article, most of the patients included in the study were treated with interferon as a first line treatment and not with anti-angiogenic drugs currently in clinical use, and in this case PFS was not analyzed and only the combination with apolipoprotein A2 (ApoA2) was predictive for OS. Their second study, which demonstrated highly significant predictive value for SAA in RCC metastatic patients treated by tyrosine-kinase inhibitors (TKI), is in agreement with our data, albeit they did not specify the SAA variant.

Another soluble molecule of interest is CFB whose expression correlated with survival and metastasis in the UroCCR cohort. Plasma measurements showed that, similarly to SAA2, patients with metastases had higher CFB plasma levels compared to patients without metastases. This remained the case whether the samples were taken before or after surgery, suggesting that CFB may be a useful blood borne marker of metastasis. Like SAA2, CFB plasma levels were tested in patients with metastasis undergoing targeted therapy, before and after a first cycle treatment with sunitinib. Patients whose CFB levels were increased following treatment had faster disease progression and shortened survival compared to patients whose levels were decreased. The combination of SAA2 and CFB plasma level measurements before treatment produced a more powerful analysis than either marker alone. This indicates that a combined analysis of these two markers may represent a tool for predicting patient outcome following targeted treatment and for therapy decision. Noteworthy, CFB is a prognostic preoperative marker in pancreatic carcinoma which outperformed CA19–9 and CEA [40]. However, the combination of CFB and SAA2 was not tested in this study.

The International Metastatic RCC Database Consortium Risk Model for metastatic RCC (IMDC) is used to predict survival in patients with metastatic RCC, treated with systemic therapy. Unfortunately, the IMDC score in the TORAVA, SUVEGIL and TCGA-KIRC cohorts was not available and could not be included in our analysis.

The combination CFB and SAA2 was a predictor for overall survival when analyzed in data set [13] from patients undergoing immunotherapy with check-point inhibitors. It would be interesting to evaluate the predictive value in patients treated with a combination of anti-angiogenic therapy and immunotherapy.

In addition to the results reported above, we provide herein a computational model that was able to describe accurately the DMFS curves observed in the clinical data. With the model, we confirmed the impact of CFB and SAA2 in metastatic dissemination and derived a predictive tool to determine the TTR. Importantly, predictions made by the mathematical model were similar to classical multivariable Cox regression (C-index = 0.73 vs 0.74 in models with Fuhrman grade and CFB). However, such a mathematical model with mechanistic basis provides added value compared to agnostic statistical tools from either classical survival analysis or machine learning. This utility is twofold. First, due to its biological ground, the model is able to distinguish the impact of biomarkers on either growth (parameter α) or dissemination (parameter μ). Second, the model is able to provide individual simulations of the disease development that could be further applied for treatment personalization (e.g., adapting the number of cycles of adjuvant therapy).

## Conclusion

All in all, we described herein a series of systematic studies to characterize the molecular events occurring at various step of tumor progression in RCC, aimed at unraveling clinically relevant molecular signatures and biomarkers. This includes the use of (i) a syngeneic mouse model, which permitted to work with an intact tumor microenvironment retaining a fully functional immune system, (ii) an experimental model based on three different modalities of serial tumor cell implantation, (iii) patient samples and data for clinical correlation and (iv) mathematical modelling.

Overall, our approach yielded very distinct transcriptomic and methylome profiles and signatures, which led to meaningful results for clinic translation as well as to a computational model for predicting tumor relapse.

## Materials and methods

### Cell culture

The murine renal cancer RENCA cell lines were maintained in RPMI-1640 (Eurobio) supplemented with 10% (v/v) FBS and 1% (v/v) penicillin-streptomycin, and incubated at 37 °C with 5% CO_2_. For the generation of GFP-expressing cells, a lentiviral vector (pRRLsin-MND-eGFP-WPRE) was obtained from the vectorology platform of the University of Bordeaux (Vect’UB), and used for infection of RENCA cells. Authentication of parental cell line was conducted by Microsynth on December 2020.

### Implantation,-extraction and tumor cell purification

For sub-capsular implantations, 1 × 10^5^ GFP-RENCA cells were injected under the left kidney capsule of 6 weeks old female BALB/c mice (Charles River Laboratories), whilst for intravenous injections 5 × 10^6^ cells were injected into the caudal vein. When a mouse from a group reached an endpoint, all mice from that group were sacrificed, and both primary tumors or lung metastases were collected. For tumor cell extraction and purification, tissues were cut into small pieces with a scalpel and digested with Collagenase I and Collagenase II (Liberase TL, Roche) for 1 h at 37 °C. Subsequently, digested tissues were filtered using cell strainers (100 μm, 70 μm and 40 μm) and cultured in complete RPMI-1640. Cell cultures were checked daily and passaged as necessary for around 2 weeks, until GFP-negative cells could not be detected.

GFP-RENCA purity was assessed either by fluorescence microscopy or flow cytometry using BD Accuri C6 (BD Bioscience). Finally, GFP-RENCA cell lines were collected for analysis or re-implanted into a new set of mice for the subsequent in-vivo passage.

Mice were housed in the animal facility of Bordeaux University (Animalerie Mutualisée, Université de Bordeaux, France). All animal experiments were approved by the “Ministère de l’Enseignement Supérieur, de la Recherche et de l’Innovation (MESRI)” (authorization numbers 2,016,072,015,478,042; 2,015,110,618,597,936 and 2,015,070,315,335,217), and were carried out in accordance with the approved protocols.

### RNA extraction, transcriptomic and qPCR gene expression analyses

Total RNA was extracted using the RNeasy Plus Mini Kit (#74134, Qiagen), according to the manufacturer’s protocols. For the analysis of the transcriptomic profiles of generated cancer cell lines, we used SurePrint G3 Mouse Gene Expression Microarrays (G4852A, Agilent). Instead, for Real-Time qPCR analysis, 1 μg of total RNA was reverse-transcribed using the high-capacity cDNA reverse transcription kit (Applied Biosystems, 4,368,814). Then, cDNAs were analyzed using either EurobioProbe or EurobioGreen master mix (Eurobio Scientific), and StepOne Real-Time PCR System (Applied Biosystems). Human HPRT1 or mouse α-Tubulin were used as housekeeping genes. List of primers is summarized in Supplementary Table S3.

### Elisa

Human plasma samples were collected from UroCCR cohort and analyzed by ELISA, according to the manufacturer’s protocols: human SAA2 (DLdevelop, DL-SAA2-Hu-96 T), Human CFB (Abcam, ab137973).

### Transcriptome data generation and analysis

Counts of samples by group and passages:

**Table:** 

Group	P0	P2	P3	P4	P5	P6
**Parental cell line**	2	NA	NA	NA	NA	NA
**KPT**	NA	2	2	3	5	5
**T-LM**	NA	2	3	4	6	4
**L-LM**	NA	1	5	3	4	3

From the log2 scale normalized data set, Principal Component Analysis (PCA, function prcomp of stats R package (v3.6.2) with the parameter center = T) was performed on parental cell line (P0) and each passage resulting in P0-P3, P0-P5 and P0-P6 PCA.

For the P0-P6 PCA, genes with the most important association were selected by keeping genes whose contributions were above the mean of all contributions for PC1 and PC2. This resulted in a set of 5194 genes. The mean value was computed for each passage P0 to P6 and used for the heatmap using the pheatmap R package (v1.0.12, clustering_method = “ward. D2”, scale = “row”).

Differential Expression Analysis (DEA) was performed between the passage 3 and 6 for each group separately (KPT, T-LM, K-LM) with the limma R package (v.3.42.2 [41]). A gene was considerate as a Differentially Expressed Gene (DEG) if its adjusted *p*-value was ≤0.01 (Benjamini & Hochberg (BH) method (CIT4)). The results are summarized in the following table.

**Table:** 

	KPT	T-LM	K-LM
**Up-regulated DEG**	454	714	223
**Down-regulated DEG**	210	145	92
**Total DEG**	664	859	315

Then, enrichment analysis was done for DEG sets of each group separately. To perform enrichment analyses we used hypergeometric test (enricher function of clusterProfiler R package [42]; v3.10.1) with go_terms.mgi downloaded on Mouse Genome Database (MGD) at the Mouse Genome Informatics website (URL: http://www.informatics.jax.org [43] (04/2019). A GO term was considered significantly enriched if its adjusted *p*-value was ≤0.05 (BH method). Then, we computed a z-score value as an additional indicator of the direction of the dis-regulation of the GO term as: z-score = (up−down)/sqrt(count) where up / down are the number of assigned genes up- or down-regulated, respectively, in the GO term [44]. Finally, we searched for commons GO terms between each possible combination of KPT, T-LM and K-LM groups. The top 5 GO terms were selected for each group and combined group based on the gene counts and the z-score.

### Biomarker strategy

Using the transcriptomics data obtained from our cell lines, we generated a list of highly differentially and progressively expressed genes. This list corresponded to the intersection of the gene sets selected as described in the steps (i) and (ii).

(i) Highly differentially expressed genes between P0 and P6.

To select genes with the highest differential expression between the parental cell line and the P6 passage, we used a z-score approach. First, we computed the log fold change (logFC) for each gene, and then the mean and standard deviation of all the logFC, obtaining at the end a z-score for each gene. A gene was considered as highly differentially expressed if its absolute z-score value was ≥2.58 (corresponding to a *p*-value of 0.01) and if its absolute logFC was ≥2.

(ii) Genes with progressive expression patterns.

We captured genes with a progressive expression pattern following the same direction during all passages with the limma R package. For this, we identified DEG (adjusted p-value ≤0.01) for each following comparison: P0-P6, P0-P2, P2-P3, P3-P4, P4-P5 and P5-P6. A gene was considered as progressive if it was differentially expressed for the P0-P6 comparison and if the direction of its differential expression was the same through all other comparisons. Stable states with no significant difference between P0-P2, P2-P3, P3-P4, P4-P5 and P5-P6 comparisons were allowed. To ensure that the genes were specific to each generated cell line, we then selected only progressive genes through all passages and late passages, by keeping progressive genes differentially expressed in P0-P6, P0-P6 and P4-P5, and P0-P6 and P5-P6.

Next, from our selected highly differentially and progressively expressed gene list, we converted *Mus Musculus* gene names to Human gene names using the conversion table from Biomart website (https://www.ensembl.org [45]).

Subsequently, we generated potentially clinically relevant gene signatures using the TCGA-KIRC cohort, as described in the following steps (iii) and (iv).

(iii) Selection of prognostic human genes.

For each gene, the KIRC cohort was segregated in 3 groups based on the expression. Then, we fitted a Cox proportional hazard regression model based on overall survival (OS) and disease-free survival (DFS) time. The cox proportional and log rank hazard ratio (HR) values were computed according with the differential expression (up or down) of the gene identified in the previous steps. A gene was selected if its HR was ≥2 and if the adjusted *p*-value (BH method) of its log-rank test was ≤0.01 for OS or DFS.

(iv) Aggregation of relevant genes as signatures.

To measure the clinical relevance of the resulting signatures, we used the SigCheck R package (v2.18, [46]). To separate samples into groups, we computed a score for each sample which corresponded to the mean value over all the expression values in the signature (scoreMethod = “High” in the sigCheck function). Patients were then ranked by their scores and split in 3 groups (high, medium, low) to perform a log rank test and compute associated HR. Signatures were tested for OS of all patients and DFS of only non-metastatic M0 patients.

(v) Validation of the signatures.

We validated the significance of each signature after adjustment for clinical variables (Fuhrman grade and TNM stage) with a multivariate Cox regression analysis (ggforest function). To show that the signatures were significantly more associated with OS and DFS outcomes than random predictors, we compared the performance of each signature with 1000 signatures composed of the same number of randomly-selected genes (sigCheckRandom function).

### TCGA KIRC cohort

TCGA Kidney Renal Clear Cell Carcinoma (KIRC) HiSeqV2 data were downloaded from XenaBrowser [47]. We chose log2(x + 1) transformed RSEM normalized count (version 2017-10-13) as recommended on its web site (https://xenabrowser.net/; page: “dataset:gene expression RNAseq-IlluminaHiSeq percentile”). We removed genes whose expression had null values in more than 2 third of all samples (2360 genes). Complementary associated clinical data were downloaded from cbioportal (www.cbioportal.org/, [48]). Only primary tumor samples were used remaining to 533 patients whose 352 had a M0 status.

### Human patient samples

Patient samples (tumor tissue and plasma) from the UroCCR cohort were used with associated clinical data (clinicaltrial.gov, NCT03293563). Eligible patients for SUVEGIL and TORAVA trials were at least 18 years of age and had metastatic ccRCC histologically confirmed, with the presence of measurable disease according to Response Evaluation Criteria in Solid Tumors v1.1.

For SUVEGIL and TORAVA cohorts, patients did not received previous systemic therapy for RCC, and were eligible for sunitinib or bevacizumab treatment in the first-line setting. Patients were ineligible if they had symptomatic or uncontrolled brain metastases, an estimated lifetime less than 3 months, uncontrolled hypertension or clinically significant cardiovascular events (heart failure, prolongation of the QT interval), history of other primary cancer. All patients gave written informed consent. Tumors were assessed at baseline and then every 12 weeks by thoracic, abdominal, pelvic and bone CT scans. Brain CT scans were performed in case of symptoms. This cohort includes patients from the SUVEGIL (24 patients) and TORAVA (35 patients) trials. The SUVEGIL trial (clinicaltrial.gov, NCT00943839) was a multi-center prospective single-arm study. The goal of the trial is to determine whether a link exists between the effectiveness of therapy with sunitinib malate and development of blood biomarkers in patients with kidney cancer. Patients received oral sunitinib (50 mg per day) once daily for 4 weeks (on days 1 to 28), followed by 2 weeks without treatment. Courses repeat every 6 weeks in the absence of disease progression or unacceptable toxicity. The TORAVA trial (clinicaltrial.gov, NCT00619268) was a randomized prospective study. Patient characteristics and results have been previously described [43]. Briefly, patients aged 18 years or older with untreated metastatic ccRCC were randomly assigned (2: 1:  1) to receive the combination of bevacizumab (10 mg kg^− 1^ iv every 2 weeks) and temsirolimus (25 mg iv weekly) IFN-α (9 mIU i.v. three times per week), or one of the standard treatments: sunitinib (50 mg per day orally for 4 weeks followed by 2 weeks off) [49]. These studies were approved by the ethic committee at each participating center and run in agreement with the International Conference on Harmonization of Good Clinical Practice Guideline. Blood samples were collected during the inclusion visit (baseline).

### Validation of signatures in a cohort treated with immunotherapy

The relevance of the KPT, K-LM and T-LM signatures, as well as SAA2, CFB and SAA2-CFB combination were also tested in a two cohorts treated with either everolimus (mTOR inhibitor, 130 patients) or nivolumab (anti-PD-1, 181 patients) immunotherapies [13]. We used.

SigCheck R package (v2.22, [46] with sigCheck function (scoreMethod = “High”). Signatures were tested for OS and Progression-Free Survival (PFS). To show that the signatures were significantly more associated with OS and PFS outcomes than random predictors, we compared the performance of each signature with 1000 signatures composed of the same number of randomly-selected genes (sigCheckRandom function).

### Computational analysis

Machine learning analysis for right-censored data was done using the Random Survival Forest (RSF) algorithm, using the RSF implementation of the randomForestSRC R package. All RSF models were fitted using 1000 trees with the log-rank splitting rule. The impact of covariates on the DMFS curves was assessed by using the forest-averaged minimal depth (similar to the analysis done in Ref A3).

To analyze clinical DMFS data we developed a mathematical model that is fully described in Ref A4. First, we developed a mechanistic model of primary tumor growth and metastasis dissemination assuming Gompertzian kinetics for tumor growth and metastasis dissemination being proportional to the primary tumor growth. ﻿The full metastatic process was described by the following transport equation

$$\left\{\begin{array}{c}{\partial}_t{\rho}^i\left(t,v\right)+{\partial}_v\left({g}^i(v){\rho}^i\left(t,v\right)\right)= 0\\ {}{g}^i\left({V}_0\right){\rho}^i\left(t,{V}_0\right)={\mu}^i{V}_p^i(t)\\ {}{\rho}^i\left( 0,v\right)= 0\end{array}\right.$$

where the function ρ^i^(t,ν) represents the distribution of metastatic tumors with size ν at time t for the individual patient i. The growth function g^i^ is defined by gi(ν) = (α^i^-β^i^ log(ν))ν and μ^i^ is the individual metastasis dissemination rate. The primary tumor volume V^i^_p_(t) follows ${V}_p^i(t)={e}^{\left(\frac{\upalpha^i}{\upbeta^i}\left(1-{e}^{\left(-{\upbeta}^it\right)}\right)\right)}$.

Second, the individual time to recurrence TTR^i^ (considered as the time elapsed from diagnosis to the appearance of the first visible metastasis) was defined as a function of three individual parameters: TTR^i^ = f(V^i^_diag_, α^i^, μ^i^), where V^i^_diag_, α^i^ and μ^i^ are the volume at diagnosis, growth rate and metastasis dissemination rate respectively. Specifically, N_vis_(TTR^i^) = 1 with ${N}_{vis}(t)={\int}_{{\mathrm{V}}_{vis}}^{+\infty }{\uprho}^i\left(t,v\right) dv$ with V_vis_ a threshold assumed to represent minimal visible size of a tumor and taken to V_vis_ = 5 mm. in diameter. We then assumed log-normal distributions for the parameters (α^i^, μ^i^) with parameters log(α^i^) = log(α_pop_) + η^i^_(α)_ where η^i^_(α)_ ~ N(O, ω^2^_(α)_) and log(μ^i^) = log(μ_pop_) + η^i^_μ_ where η^i^_μ_ ~ N(O, ω^2^_μ_). The population distribution of TTRs allows to define the population DMFS curves as DFMS(t; α_pop_, μ_pop_, ω_a_, ω_μ_) = P[TTR > t].

We analyzed three possible effects of the most important covariates on tumor growth and metastasis dissemination and selected the effect with the lowest difference between the model predictions and the clinical data.

For a given patient, we mathematically computed 10,000 in silico replicates with identical values for the covariates FG, CFB and Saa2, and calculated the proportions of virtual patients with metastasis at diagnosis and patients without metastasis after 5 years. We defined the “predicted TTR” as the median of the 10,000 TTR. Harrell’s C-index was computed using the predicted TTR and the clinical TTR values, allowing for right-censored data. Confidence intervals were calculated bootstrapping the data 100 times and using the percentile method.

### Statistical analyses

Mann-Whitney U test and unpaired two-tail Student t-test were used for in vivo and in vitro, respectively, experiments. *p*-values < 0.05 were considered statistically significant (* *p* < 0.05; ** *p* < 0.01; *** *p* < 0.001). Statistical analyses were performed using either R studio (R v3.6.2 [50], R studio v1.2.5033 [51]) or GraphPad Prism (version 6.00 for Windows, La Jolla California USA, www.graphpad.com). Survival analysis were performed using survival ([52], v3.2–7) survminer (v0.4.8). Analysis of transcriptomics, methylomics, enrichment and signature computations were performed using R.

For Transwell migration assay, Tissues processing and immunofluorescent analysis, Low-coverage whole-genome sequencing and Methylomics data generation and analysis, see Supplementary Materials and Methods.

## Supplementary Information

**Additional file 1: Supplementary Materials and Methods**. Transwell migration assay. Tissues processing and immunofluorescent analysis. Low-coverage whole-genome sequencing. Methylomics data generation and analysis.

**Additional file 2: Supplementary Fig. S1**.

**Additional file 3: Supplementary Fig. S2**.

**Additional file 4: Supplementary Fig. S3**.

**Additional file 5: Supplementary Fig. S4**.

**Additional file 6: Supplementary Fig. S5**.

**Additional file 7: Supplementary Fig. S6**.

**Additional file 8: Supplementary Fig. S7**.

**Additional file 9: Supplementary Fig. S8**.

**Additional file 10: Supplementary Fig. S9**.

**Additional file 11: Supplementary Table S1**.

**Additional file 12: Supplementary Table S2**.

**Additional file 13: Supplementary Table S3**.

## Data Availability

Microarray gene expression data is available via Gene Expression Omnibus using the accession GSE142109 (Reviewer Access Token: klwrusmcbxkpjkz). DNA sequencing was deposited in ArrayExpress: http://www.ebi.ac.uk/arrayexpress/experiments/E-MTAB-8645, Access for reviewers: Username:Reviewer_E-MTAB-8645 Password: Eaa1yr9I. Methylation data is available via Gene Expression Omnibus using the accession GSE139338 (https://www.ncbi.nlm.nih.gov/geo/query/acc.cgi?acc=GSE139338; Reviewer Access Token: mxebousmdlyzbcp. Other data and material are available upon request.
