# Supplementary material for: Experimental and computational modeling for signature and biomarker discovery of renal cell carcinoma progression
Source: Mol Cancer. 2021 Oct 20;20:136. doi: 10.1186/s12943-021-01416-5 (PMC8527701; doi:10.1186/s12943-021-01416-5)
Supplement: Supplementary file 1 — Additional file 1: Supplementary Materials and Methods. Transwell migration assay. Tissues processing and immunofluorescent analysis. Low-coverage whole-genome sequencing. Methylomics data generation and analysis. [file 12943_2021_1416_MOESM1_ESM.pdf]

Supplementary Material and methods

Transwell migration assay

The migratory properties of tumor cells were analyzed in Transwell chamber without membrane coating (24-well insert, 8µm pore size, cat 353097, Falcon). 5×10^5 GFP-RENCA cells/insert (P0, P1 or P6) were seeded in the top chamber using serum-free medium, while the lower chamber contained complete medium with 10% FBS. After 24 hours, cells were fixed in 4% PFA (sc-281692, Santa Cruz Biotechnology) and stained with DAPI. Non-migrated cells on the top of the insert membrane were removed using a cotton swap. Number of migrated cells was analyzed by fluorescent microscopy and counted as cells per acquired field using Fiji software.

Tissues processing and immunofluorescent analysis

For GFP-RENCA purity assessment after extraction from animal tissues, cells were fixed with 4% PFA and stained with both DAPI and Alexa-Fluor 546 Phalloidin (A22283, Invitrogen) for visualization of GFP-positive and GFP-negative cells.

For histological analysis, whole murine organs were fixed in 4% PFA (sc-281692, Santa Cruz Biotechnology) for 2 hours and then incubated for 72 hours in 30% sucrose at 4°C. Then, samples were embedded with OCT Compound (4583, Tissue-Tek OCT compound, Sakura). Before embedding, lungs were inflated with 1mL of diluted OCT (1:1 PBS/OCT dilution). Finally, frozen organs were stored at -80°C.

For staining of proliferative cancer cells in primary tumors, 10µm sections were prepared using a cryostat (Leica CM1900) and furtherly fixed with 4% PFA for 15 minutes. Then, tissues were boiled for 10 minutes in sodium citrate buffer (10mM, pH6) using a microwave, and blocked with 3% BSA-PBS. Anti-Ki67 primary antibody (1:250, ab16667, Abcam) and FluoProbes® 547H Donkey Anti-Rabbit secondary antibody (FP-SB5110, Interchim) were used at RT for 2 hours or 30 minutes, respectively. Finally, cell nuclei were counterstained with DAPI.

Fluorescent microscopy was performed with a Nikon Eclipse i90 microscope (Nikon) and NIS-Elements AR 4.30 software (Nikon). A slide scanner (Hamamatsu, Nanozoomer 2.0HT) from the Bordeaux Imaging Center was used for whole slide imaging using NDP.scan software (Hamamatsu). Analysis were performed using Fiji.

Low-coverage whole-genome sequencing

Genomic DNA was isolated use the Dneasy Blood and Tissue Kit (cat 69504, Qiagen), according to the manufacturer's protocols. Libraries were created using the KAPA LTP library preparation kit (Kapa Biosystems) following the manufacturer's recommendations, and sequenced on an Illumina HiSeq4000 sequencer in 51bp single-end mode. Raw reads were mapped to the mouse reference genome (mm10/GRCm38) using the Burrows-Wheeler Aligner [1]. On average, 6,882,693 reads were mapped per sample. PCR duplicates were removed using Picard (v1.43) resulting in, on average, 5,966,979 uniquely mapped reads per sample. These reads were further processed with the Bioconductor package QDNASeq.mm10. Problematic regions were excluded and read counts were corrected for mappability using LOESS regression. The number of reads was counted in bins of 50kb along the genome, log2-transformed and normalized by the median. These resulted in logR values per bin, that were subsequently segmented using the ASCAT algorithm [2]. These segmented values, as well as the individual logR values per bin, were used to plot the individual copy-number profile of each sample. Subclonal tumor fractions of the samples were estimated by ABSOLUTE [3].

Methylomics data generation and analysis

Whole Genome Bisulfite Sequencing (WGBS) was performed for three replicates of P6 groups for KPT, T-LM and K-LM cell lines, and one for the parental P0 cell line at the GeT-PlaGe core facility (INRA Toulouse). WGBS libraries have been prepared according to Biooscientific's protocol using the Biooscientific NEXTflex™ Bisulfite Library Prep Kit for Illumina Sequencing. Briefly, DNA

was fragmented by sonication, size selection was performed using Agencourt AMPure beads XP and adaptators were ligated for sequencing. Then, bisulfite treatment was performed for 2.5 hours using the EZ DNA Methylation-Gold™ Kit from Zymo Research, and 12 cycles of PCR were performed. Library quality was assessed using an Advanced Analytical Fragment Analyser, and libraries were quantified by qPCR using the Kapa Library Quantification Kit. WGBS experiments have been performed on an Illumina HiSeq3000 using a paired end read length of 2 x 150pb with the Illumina HiSeq3000 Reagent Kits. To determine conversion efficiency, fastq files were trimmed for adapters and low quality bases with Trim Galore (v0.4.4, calling cutadapt 1.3 then mapped to the pUC19 reference genome (pUC19.fa) with Bismark (v0.13.0 [4]. Samtools (v0.1.19-44428cd [5] was used to remove duplicated reads. Then methylation calling was performed with Bismark_methylation_extractor. As methylated and non-methylated cytosine positions are known on the pUC19 reference genome, over and under-conversion could be assessed. Filtered fasq file were generated by CASAVA 2.17. Fastq files were aligned with Bismark (v0.17.1_dev) against the GRCm38.p5 mus musculus genome (download from http://www.ensembl.org/, release 89) with following parameters -N 0 and -maxins 800. Bismark use Bowtie 2 (v2.3.4.3) and samtools (v1.9). Incomplete bisulfite conversion filtering was done on Bismark BAM files in order to remove reads that exceed a certain threshold of methylated calls in non-CG context. Then, de-duplication was applied (deduplicate_bismark) followed by the extraction of methylated positions (bismark_methylation_extractor).

We then considered CG positions with at least 10 reads of coverage resulting in 106303 CGs on chromosomes 1-19 and X. PCA was performed on methylation frequencies of all CGs for P0 and P6 samples (function prcomp of stats R package (v3.6.2) with the parameter center = T).

Supplementary Fig. Legends

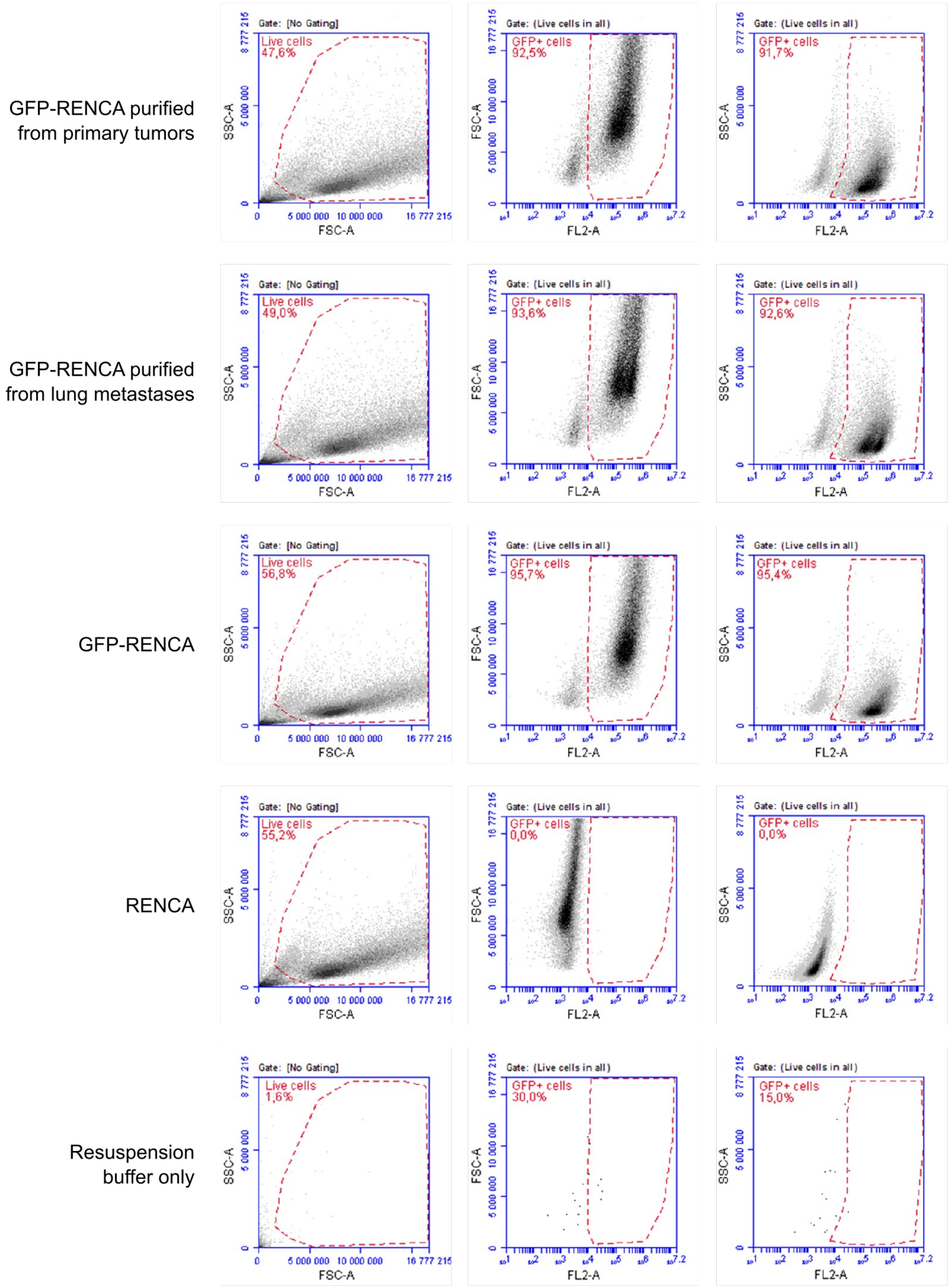

Supplementary Fig. S1. Flow cytometry analysis to assess GFP-RENCA purification. After

isolation from primary tumors or lung metastases, GFP-RENCA cells were maintained in culture for 14 days in complete medium, and finally analyzed using the BD Accuri C6 cytometer. Forward and side scatter area (FSC-A and SSC-A, respectively) plots were used to gate live cells. Gated cells were then plotted using FL2-A and FSC-A (or SSC-A) values to measure the percentage of GFP positive cells. As control, both GFP positive and negative parental RENCA cells were used to establish a maximum purity value.

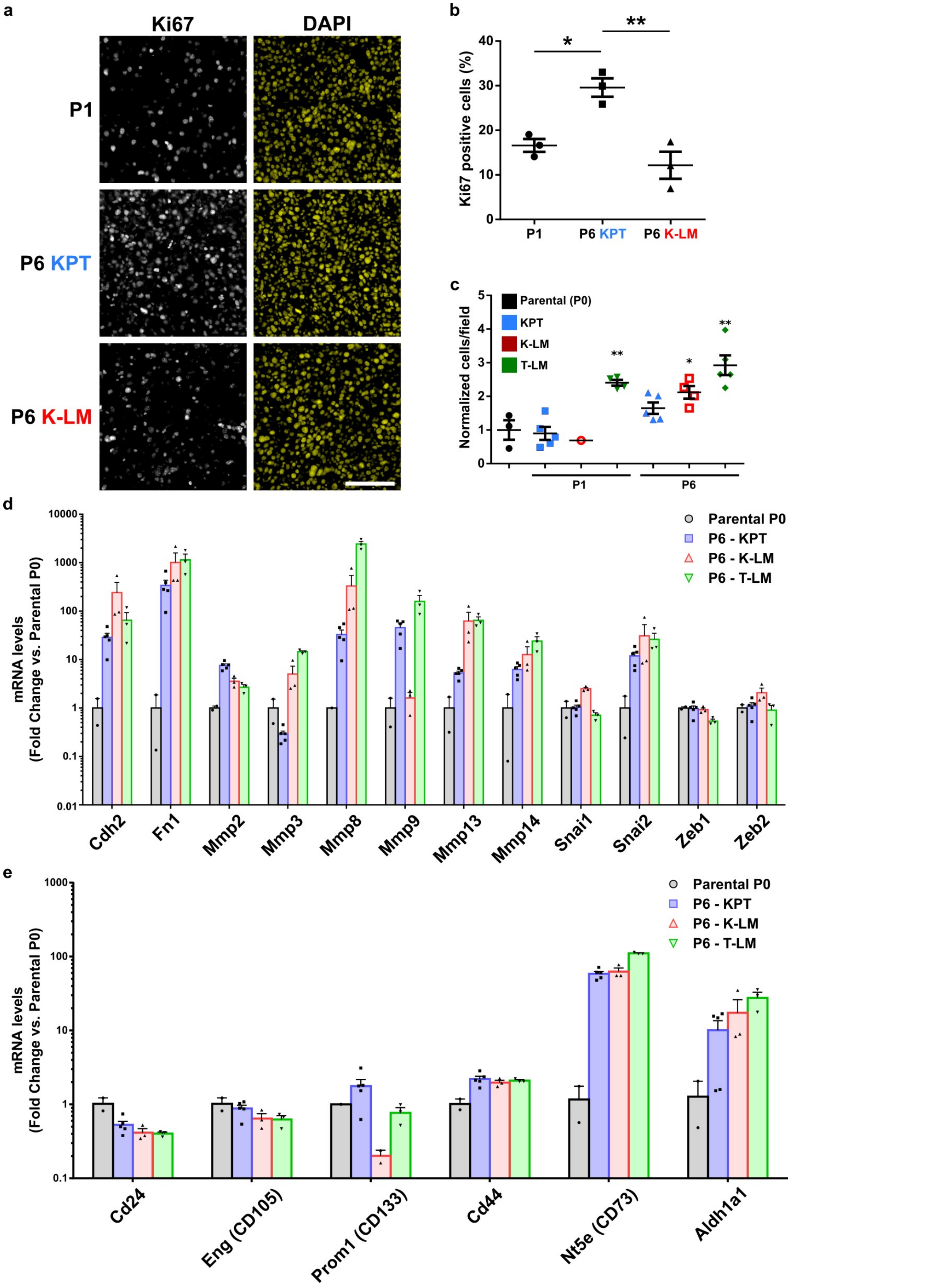

Supplementary Fig. S2. Characterization of the P6 cell lines. **a** Ki67 staining of P1 and P6 primary tumors, scale bar=100µm. **b** quantification of Ki67 positive cells (n=3 animals/group). **c**,

Quantification of the Transwell migration assay as number of migrated cells per field. (n= 1-5 biological replicate). **d** qPCR analysis of EMT markers in parental P0 and late passaged P6 cell lines. **e** qPCR analysis of RCC cancer stem cell markers in parental P0 and late passaged P6 cell lines. Data are shown as mean ± SEM.

Original ID	Sequencing ID	Number of mapped reads	Number of mapped reads after duplicate removal	percentage of duplication
RCC_P6K1	sBIK_RCC_P6K1	7,610,009	6,700,571	12%
RCC_P6K2	sBIK_RCC_P6K2	8,039,256	6,949,649	14%
RCC_P6K3	sBIK_RCC_P6K3	1,478,913	1,320,011	11%
RCC_P6K4	sBIK_RCC_P6K4	9,210,177	7,947,579	14%
RCC_P6K5	sBIK_RCC_P6K5	8,834,963	7,649,950	13%
RCC_P6L1	sBIK_RCC_P6L1	3,753,068	3,231,821	14%
RCC_P6L2	sBIK_RCC_P6L2	3,159,112	2,767,040	12%
RCC_P6L3	sBIK_RCC_P6L3	3,018,133	2,619,286	13%
RCC_P6T2	sBIK_RCC_P6T2	10,002,485	8,703,020	13%
RCC_P6T3	sBIK_RCC_P6T3	7,733,040	6,653,514	14%
RCC_P6T4	sBIK_RCC_P6T4	12,891,053	11,192,992	13%
RCC_P6T5	sBIK_RCC_P6T5	3,400,452	3,014,616	11%
RCC_Parental	sBIK_RCC_Parental	10,344,347	8,820,686	15%

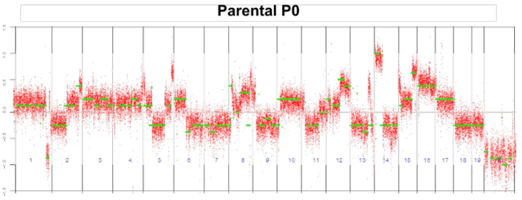

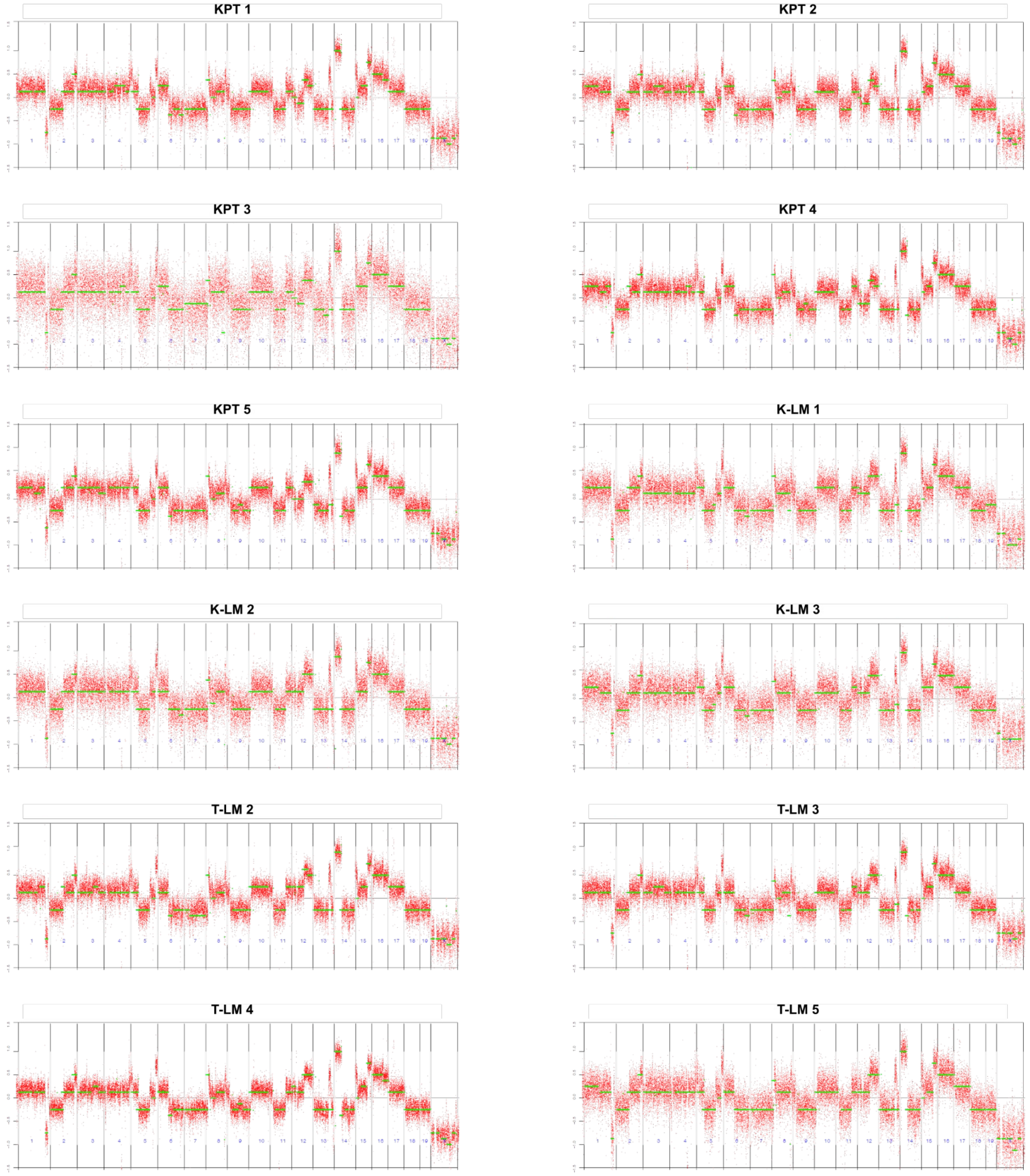

Supplementary Fig. S3. Low-coverage shallow DNA sequencing of the P6 versus P0 cells. Five

KPT, three K-LM and four T-LM cell lines were sequenced. No significant copy number variation was observed among the different groups (19% for parental versus 18%±1% for passaged lines). Visually, we also failed to observe new copy number events. When using ABSOLUTE to estimate purity (proportion of cancer cells present in the sample) and mean ploidy of the samples, no differences were noted (purity and ploidy in parental line were of 63% and 2.69, respectively versus 64%±2% and 2.65±3% for passaged lines). Thus, this indicates that the transcriptomic changes that we observed in the generated cell lines were mainly due to epigenetic adaptations.

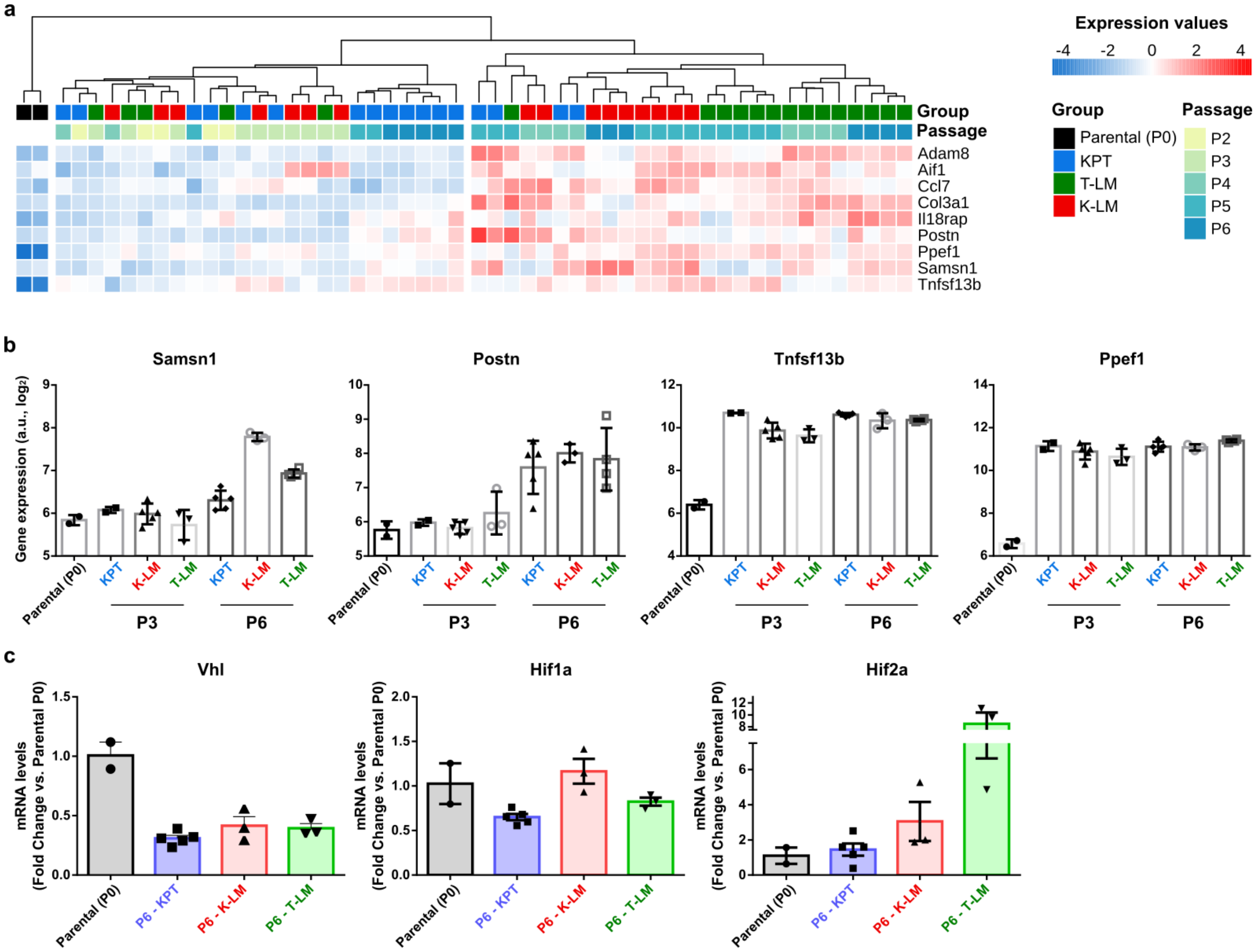

Supplementary Fig. S4. Expression profile analysis of VHL-KO RENCA up-regulated genes in our generated cell lines. **a** The heatmap describes expression changes, occurred in our cell lines, of the 9 genes identified by Schokrpur et al. [6] as significantly up-regulated upon VHL depletion in RENCA cells. The gene Cuzd1 was not measured in our data set. Hierarchical clustering shows three main different clusters. **b** Graphs show gene expression Microarrays analysis of four HIF1α target genes (i.e. Samsn1, Postn, Tnfsf13b and Ppef1). To note, these genes are poor prognostic factors in RCC patients. **c** qPCR analysis of Vhl, Hif1a and Hif2a in parental P0 and late passaged P6 cell lines. Data are represented as mean ± SD. a.u., arbitrary unit.

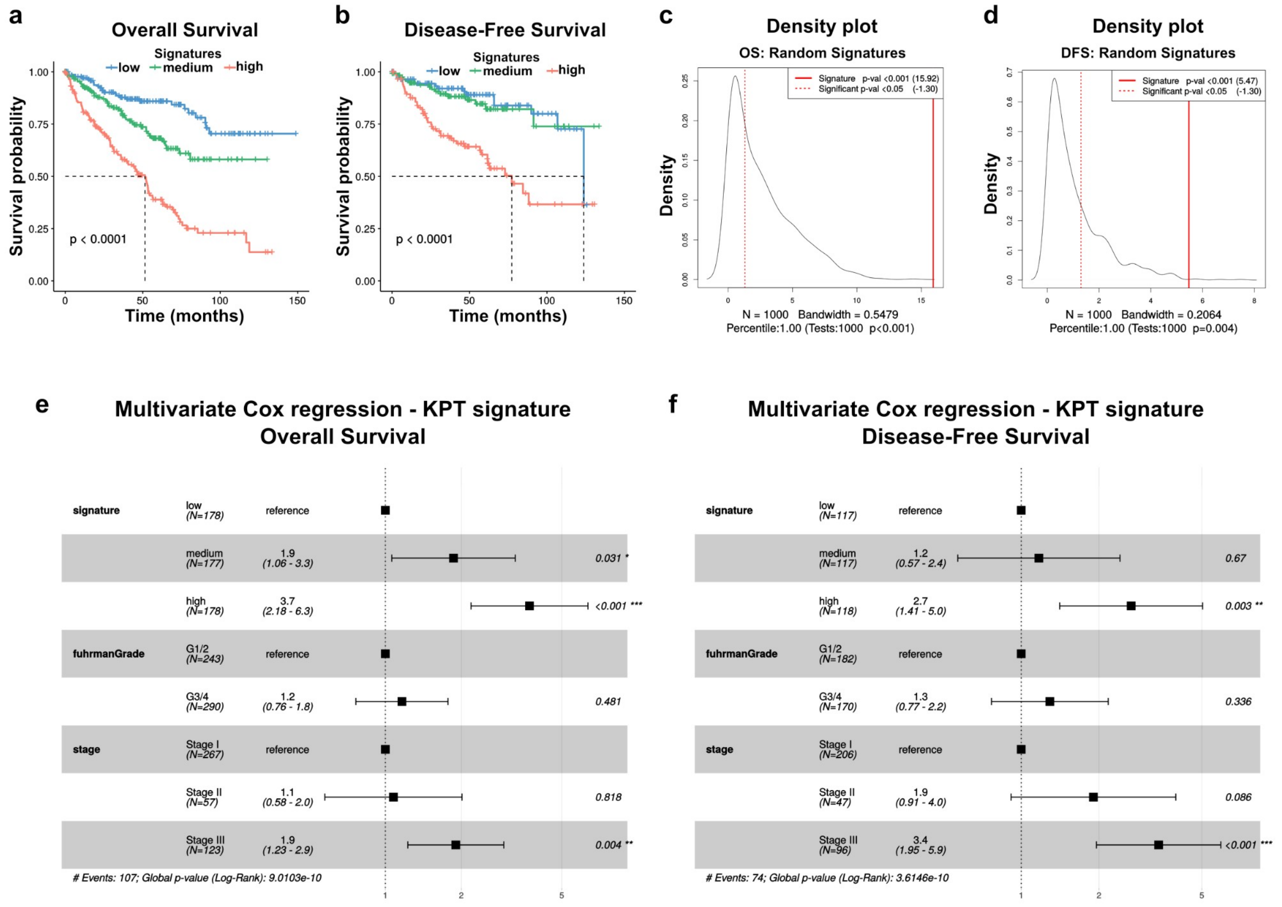

Supplementary Fig. S5. Validation of the KPT signature in the KIRC-TCGA cohort. **a** and **b** Kaplan-Meier for Overall Survival (OS) and Disease-Free Survival (DFS) analysis stratified in 3 groups of equivalent size based on the signature score. "low": patient group with low score; "medium": patient group with a medium score; "high": patient group with high score. **c** and **d** Density plot of p values (log-rank test) from 1000 random signatures of equal size for OS and DFS. p = empirical p-value. **e** and **f** Forest plot of multivariate Cox proportional hazards model for the KPT signature in the KIRC cohort adjusted for clinical variables (Fuhrman grade and TNM stage).

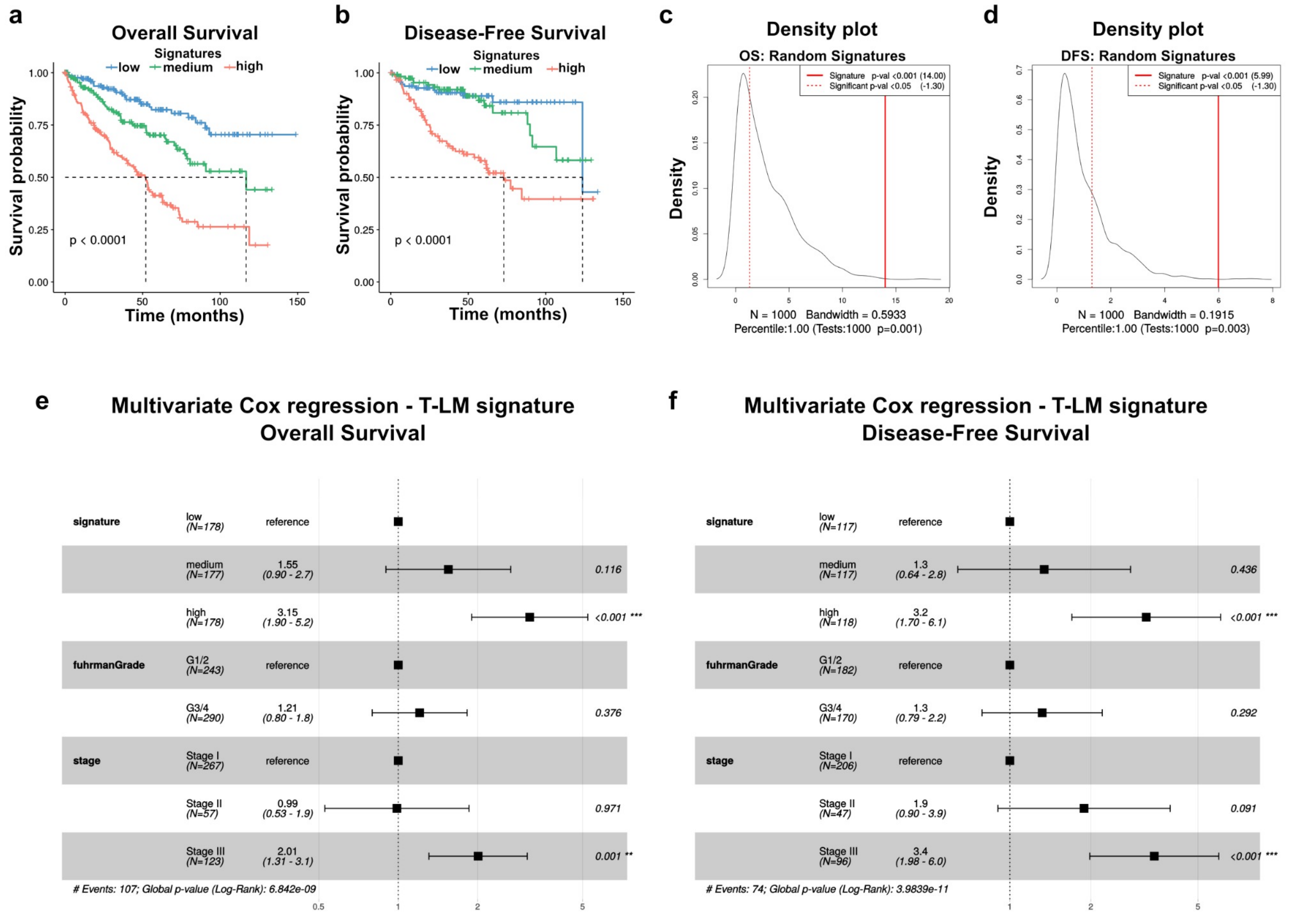

Supplementary Fig. S6. Validation of the T-LM signature in the KIRC-TCGA cohort. **a** and **b** Kaplan-Meier for Overall Survival (OS) and Disease-Free Survival (DFS) analysis stratified in 3 groups of equivalent size based on the signature score. "low": patient group with low score; "medium": patient group with a medium score; "high": patient group with high score. **c** and **d** Density plot of p values (log-rank test) from 1000 random signatures of equal size for OS and DFS. p = empirical p-value. **e** and **f** Forest plot of multivariate Cox proportional hazards model for the T-LM signature in the KIRC cohort adjusted for clinical variables (Fuhrman grade and TNM stage).

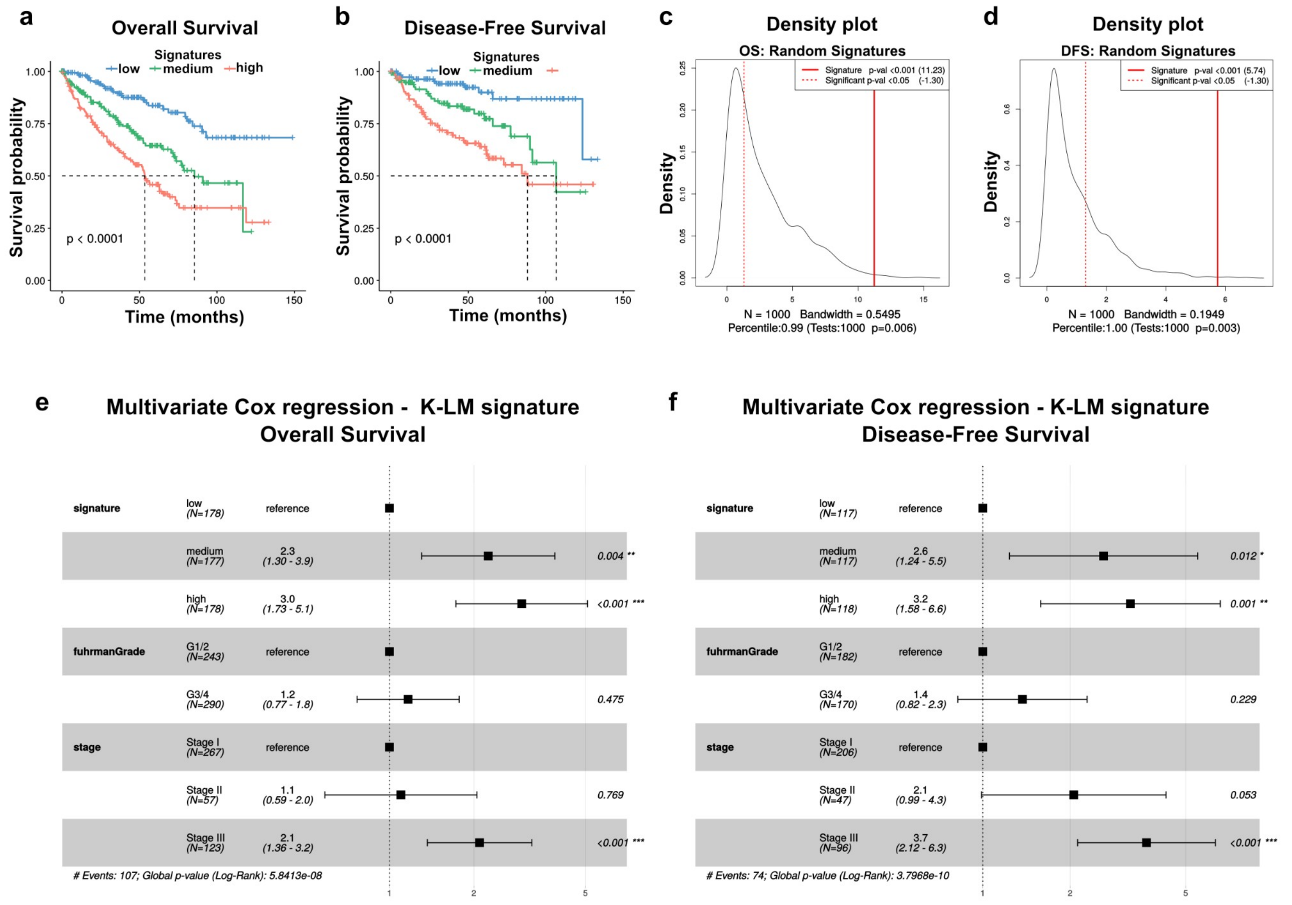

Supplementary Fig. S7. Validation of the K-LM signature in the KIRC-TCGA cohort. **a** and **b** Kaplan-Meier for Overall Survival (OS) and Disease-Free Survival (DFS) analysis stratified in 3 groups of equivalent size based on the signature score. "low": patient group with low score; "medium": patient group with a medium score; "high": patient group with high score. **c** and **d** Density plot of p values (log-rank test) from 1000 random signatures of equal size for OS and DFS. p = empirical p-value. **e** and **f** Forest plot of multivariate Cox proportional hazards model for the Lung K-LM signature in the KIRC cohort adjusted for clinical variables (Fuhrman grade and TNM stage).

a (KPT)

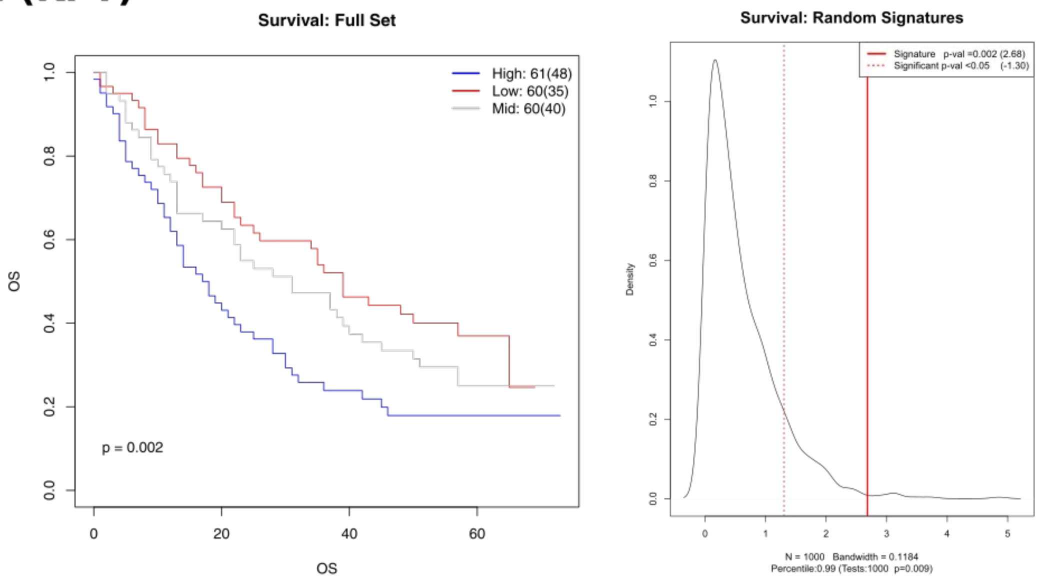

b (T-LM)

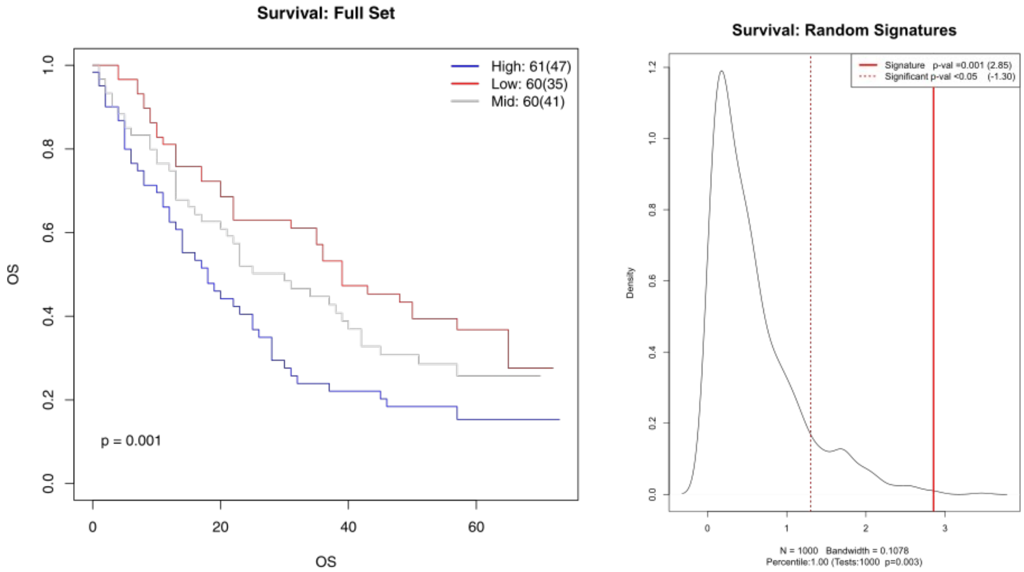

c (K-LM)

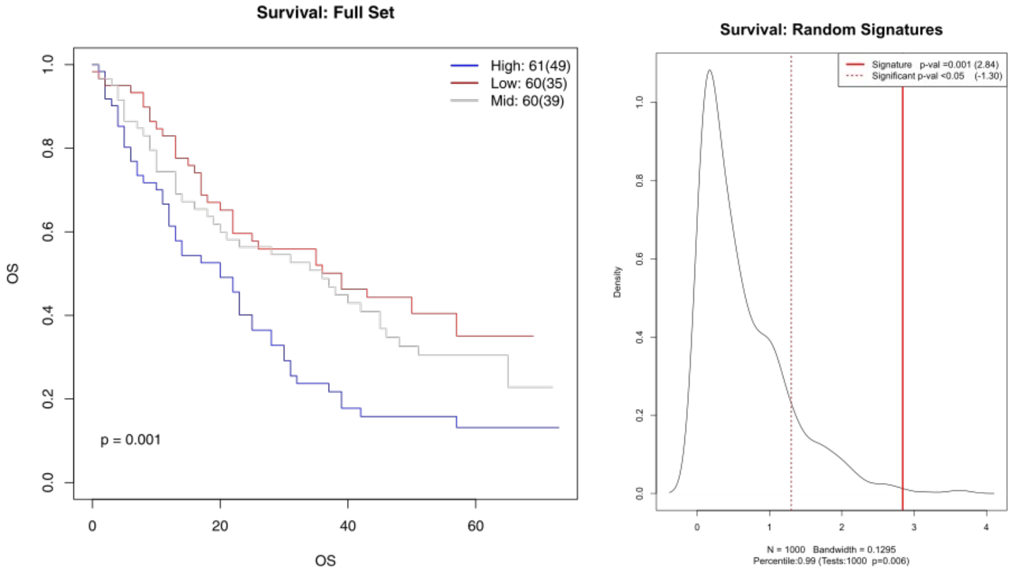

Supplementary Fig. S8. Signatures in the Nivolumab cohort. The KPT, T-LM, K-LM signatures are analyzed in the patient cohort treated with Nivolumab (left panels from top to bottom) for OS. As control, 1000 randomly-selected signatures are also tested (right panels, from top to bottom).

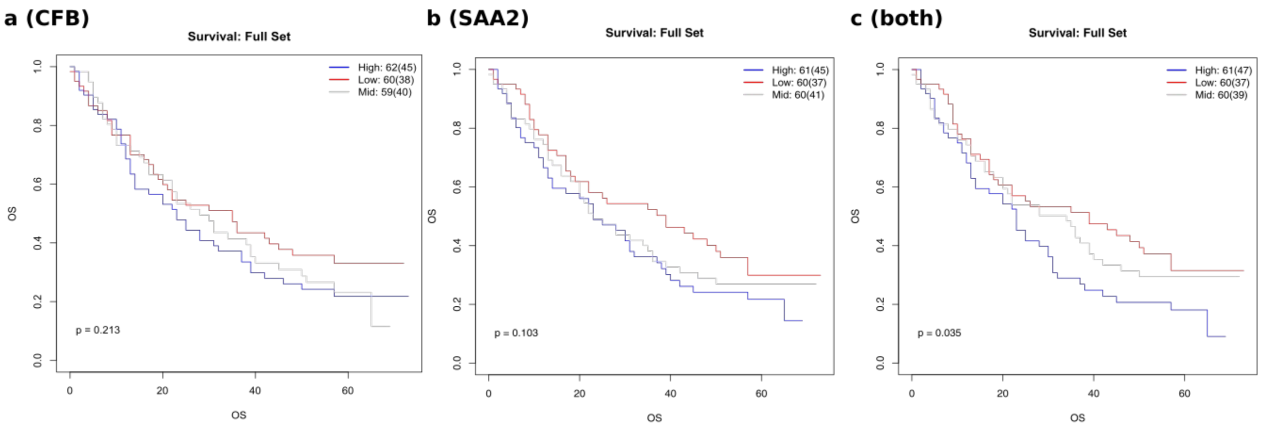

Supplementary Fig. S9. CFB and SAA2 analyzed in the immunotherapy cohort

Supplementary Table S1. (Suppl table 1.xls)

Supplementary Table S2. (Suppl table 2.xls)

Supplementary Table S3. Primers list **(Suppl table 3.xls)**

Supplementary Bibliography

1. Li H, Durbin R. Fast and accurate short read alignment with Burrows-Wheeler transform. Bioinformatics [Internet]. 2009 [cited 2019 Nov 28];25:1754–60. Available from: https://academic.oup.com/bioinformatics/article-lookup/doi/10.1093/bioinformatics/btp324

2. Van Loo P, Nordgard SH, Lingjærde OC, Russnes HG, Rye IH, Sun W, et al. Allele-specific copy number analysis of tumors. Proc Natl Acad Sci U S A [Internet]. 2010 [cited 2019 Nov 28];107:16910–5. Available from: http://www.pnas.org/cgi/doi/10.1073/pnas.1009843107

3. Carter SL, Cibulskis K, Helman E, McKenna A, Shen H, Zack T, et al. Absolute quantification of somatic DNA alterations in human cancer. Nat Biotechnol [Internet]. 2012 [cited 2019 Nov 28];30:413–21. Available from: http://www.nature.com/articles/nbt.2203

4. Krueger F, Andrews SR. Bismark: A flexible aligner and methylation caller for Bisulfite-Seq applications. Bioinformatics. 2011;27:1571–2.

5. Li H, Handsaker B, Wysoker A, Fennell T, Ruan J, Homer N, et al. The Sequence Alignment/Map format and SAMtools. Bioinformatics. 2009;25:2078–9.

6. Schokrpur S, Hu J, Moughon DL, Liu P, Lin LC, Hermann K, et al. CRISPR-Mediated VHL Knockout Generates an Improved Model for Metastatic Renal Cell Carcinoma. Sci Rep [Internet]. Nature Publishing Group; 2016 [cited 2021 May 20];6. Available from: https://pubmed.ncbi.nlm.nih.gov/27358011/
