# Supplementary figures and images for: Experimental and computational modeling for signature and biomarker discovery of renal cell carcinoma progression
Source: Mol Cancer. 2021 Oct 20;20:136. doi: 10.1186/s12943-021-01416-5 (PMC8527701; doi:10.1186/s12943-021-01416-5)

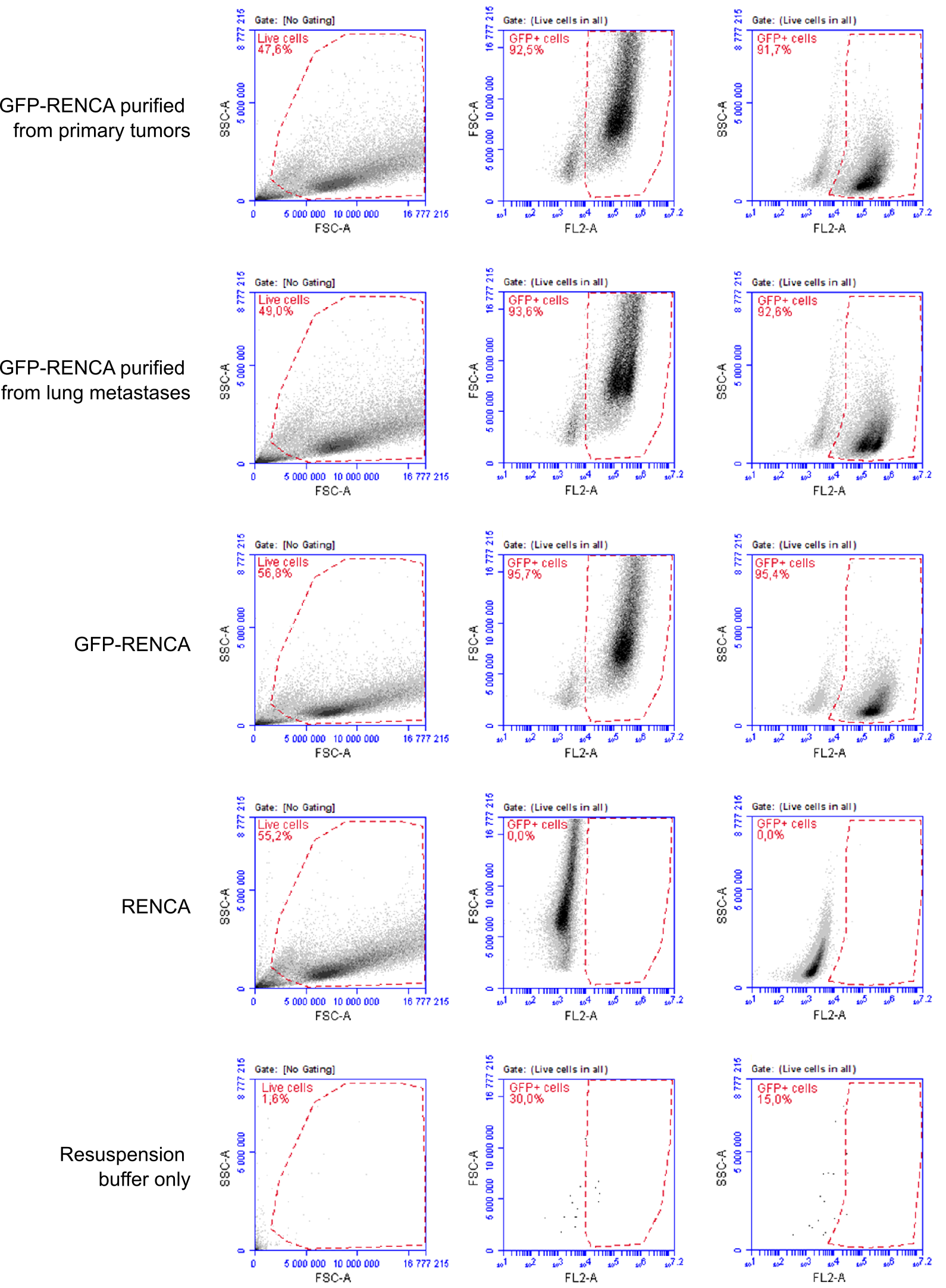

Supplement: Supplementary file 2 — Additional file 2: Supplementary Fig. S1. [file 12943_2021_1416_MOESM2_ESM.png]

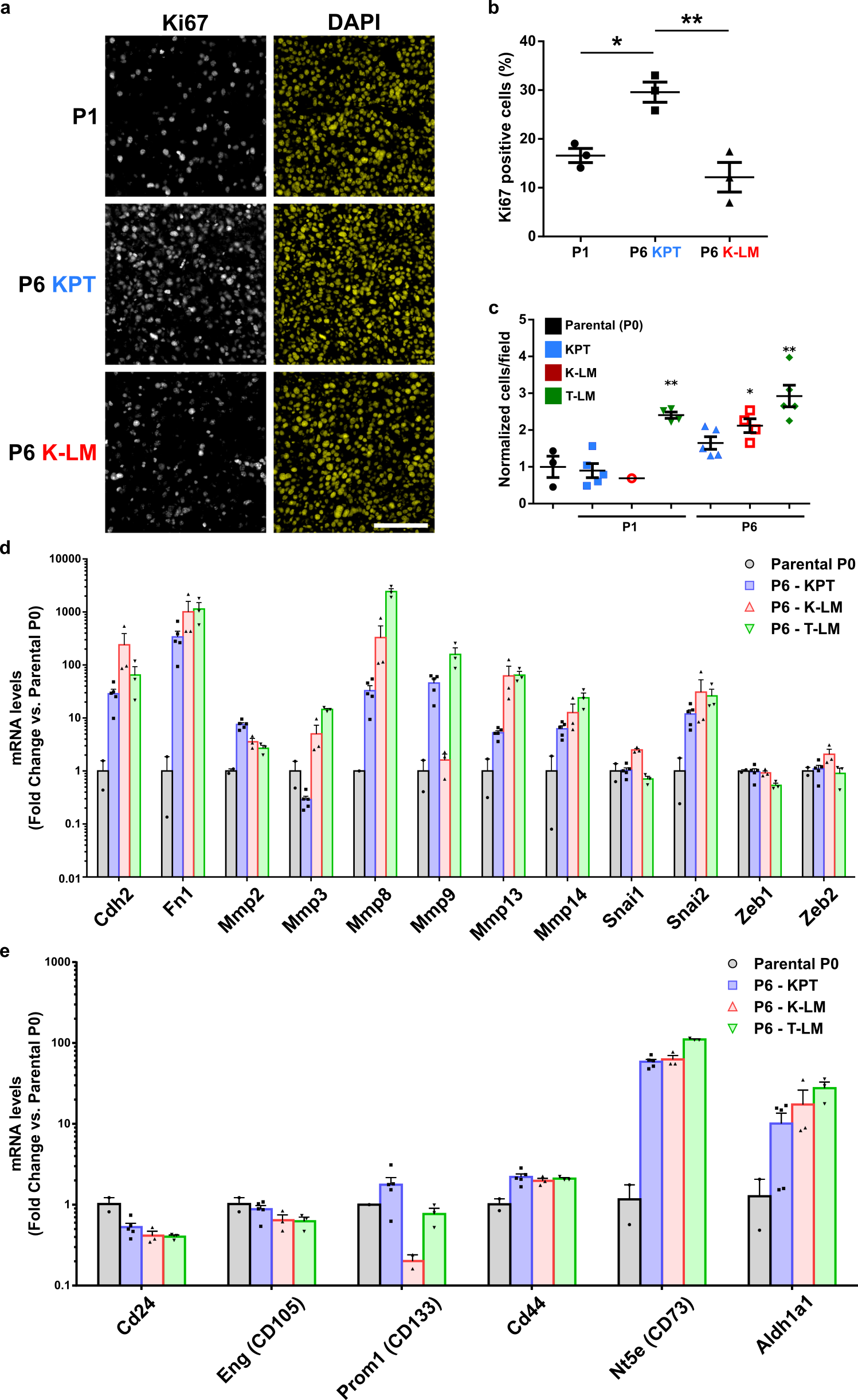

Supplement: Supplementary file 3 — Additional file 3: Supplementary Fig. S2. [file 12943_2021_1416_MOESM3_ESM.png]

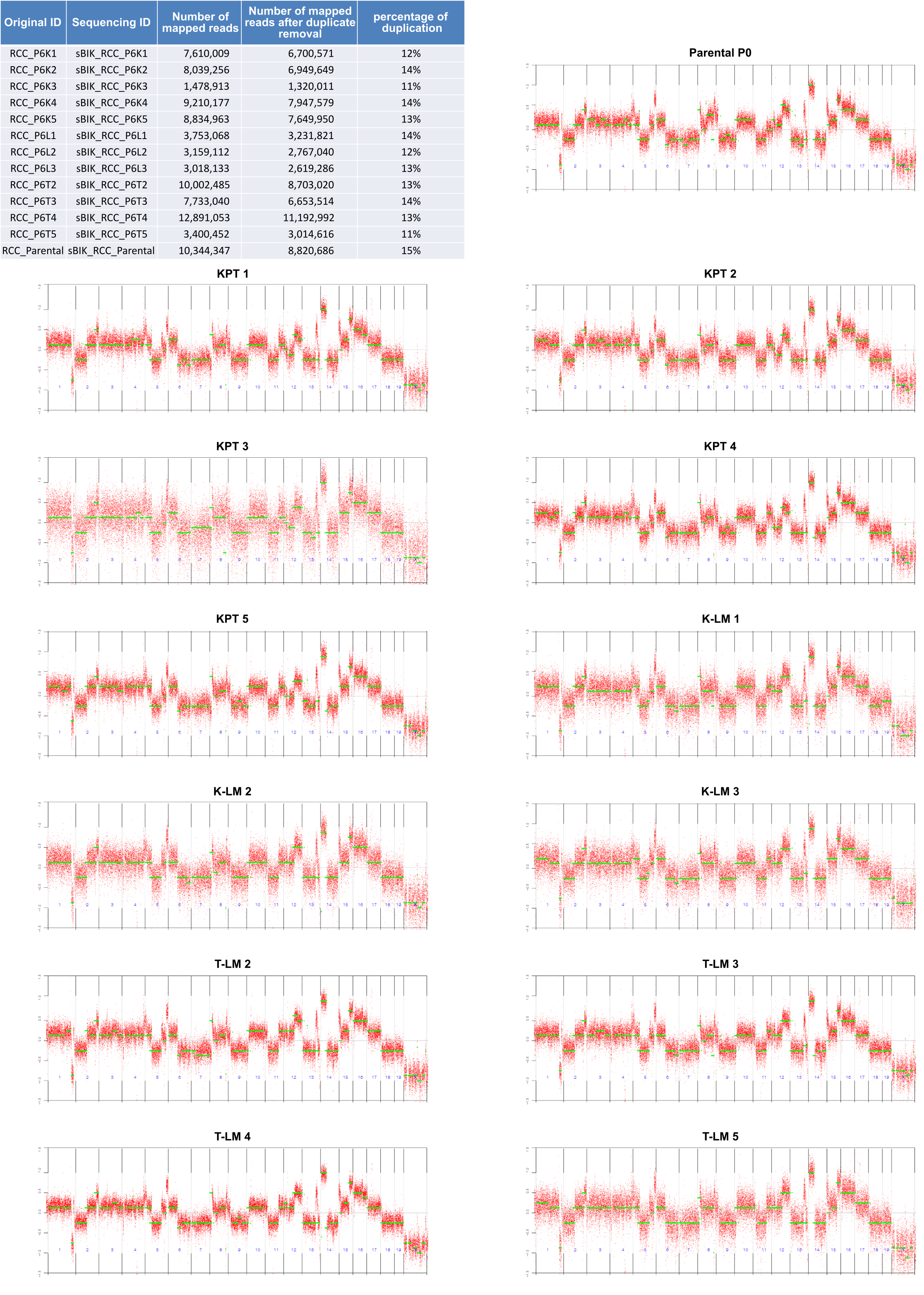

Supplement: Supplementary file 4 — Additional file 4: Supplementary Fig. S3. [file 12943_2021_1416_MOESM4_ESM.png]

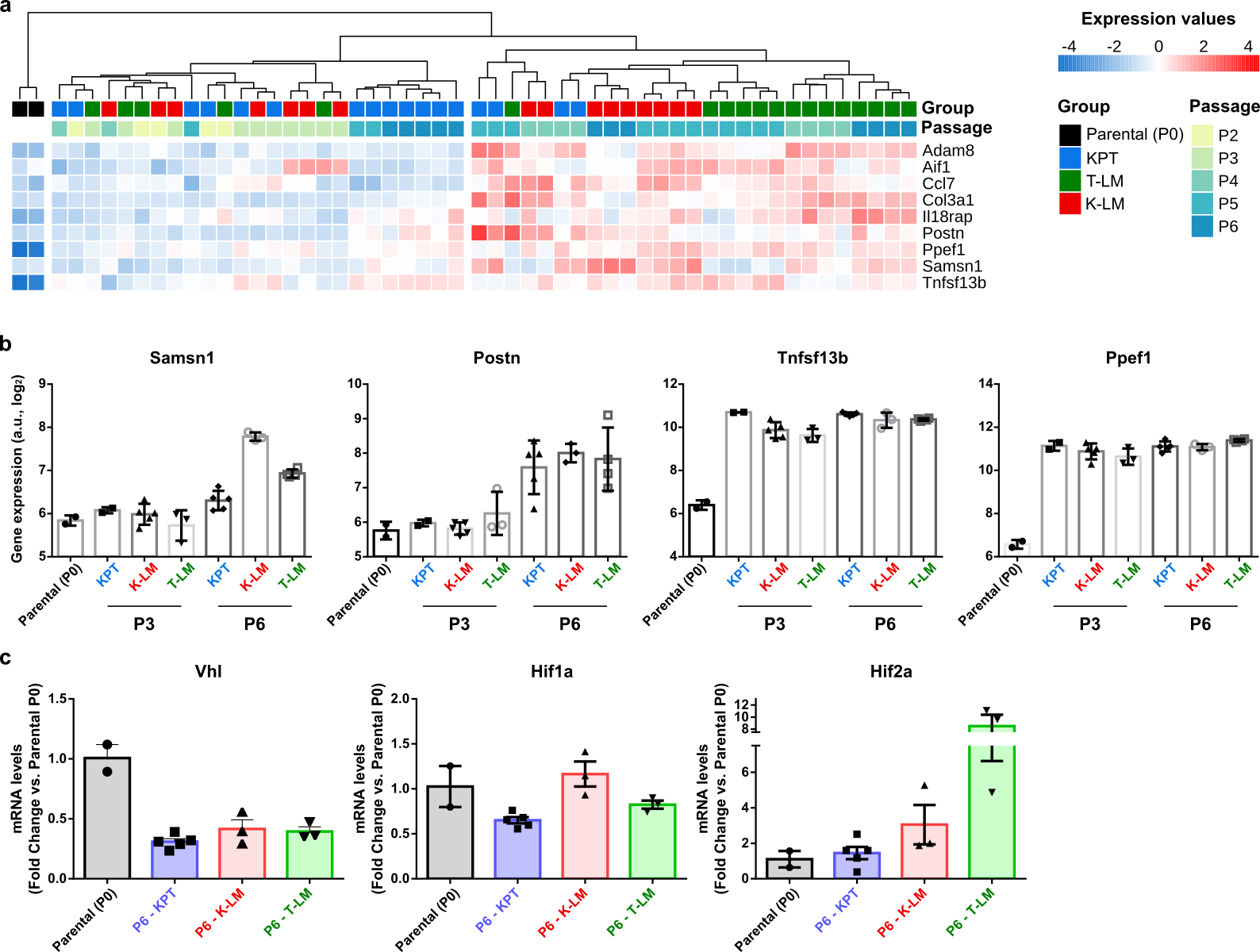

Supplement: Supplementary file 5 — Additional file 5: Supplementary Fig. S4. [file 12943_2021_1416_MOESM5_ESM.png]

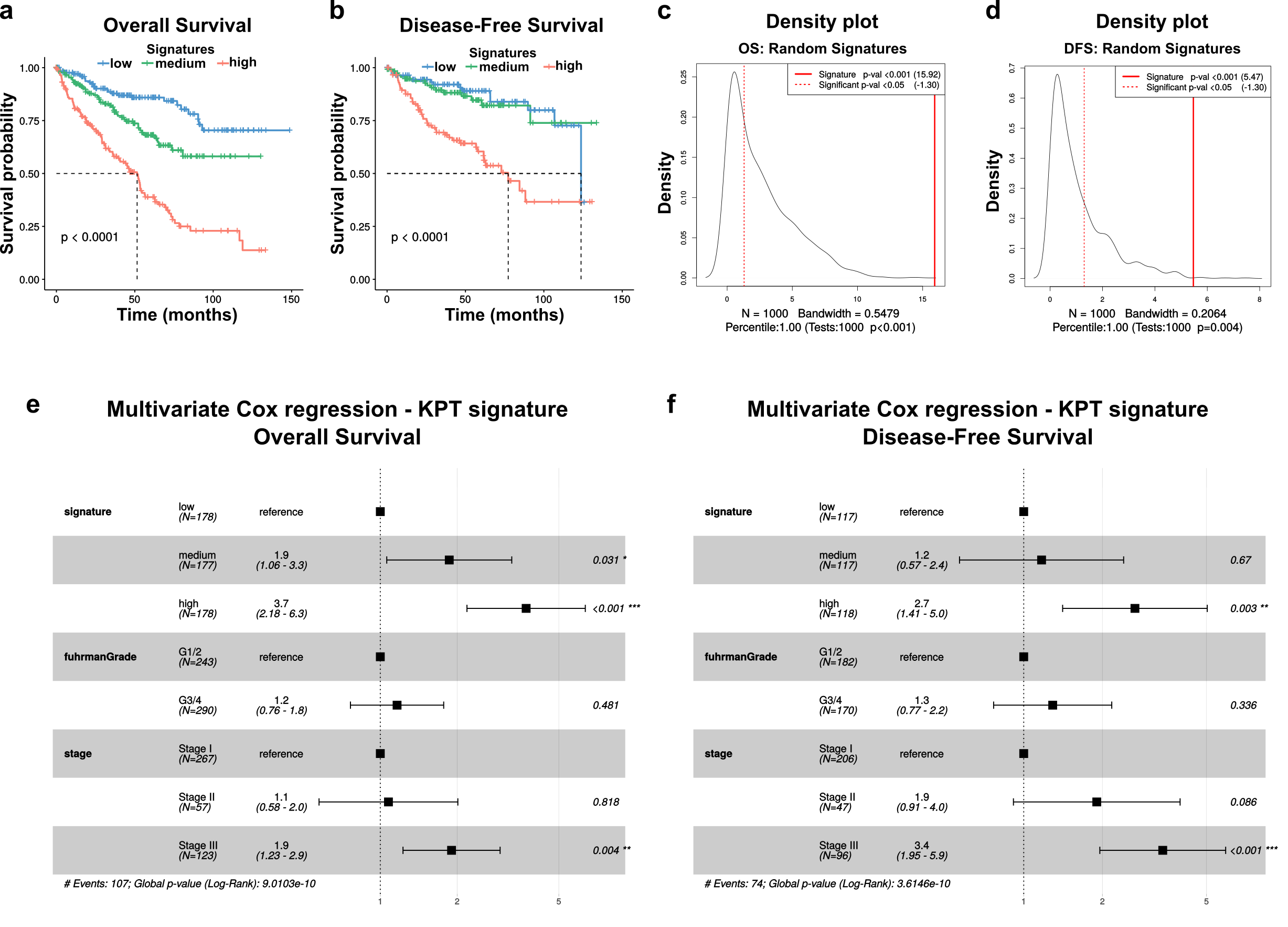

Supplement: Supplementary file 6 — Additional file 6: Supplementary Fig. S5. [file 12943_2021_1416_MOESM6_ESM.png]

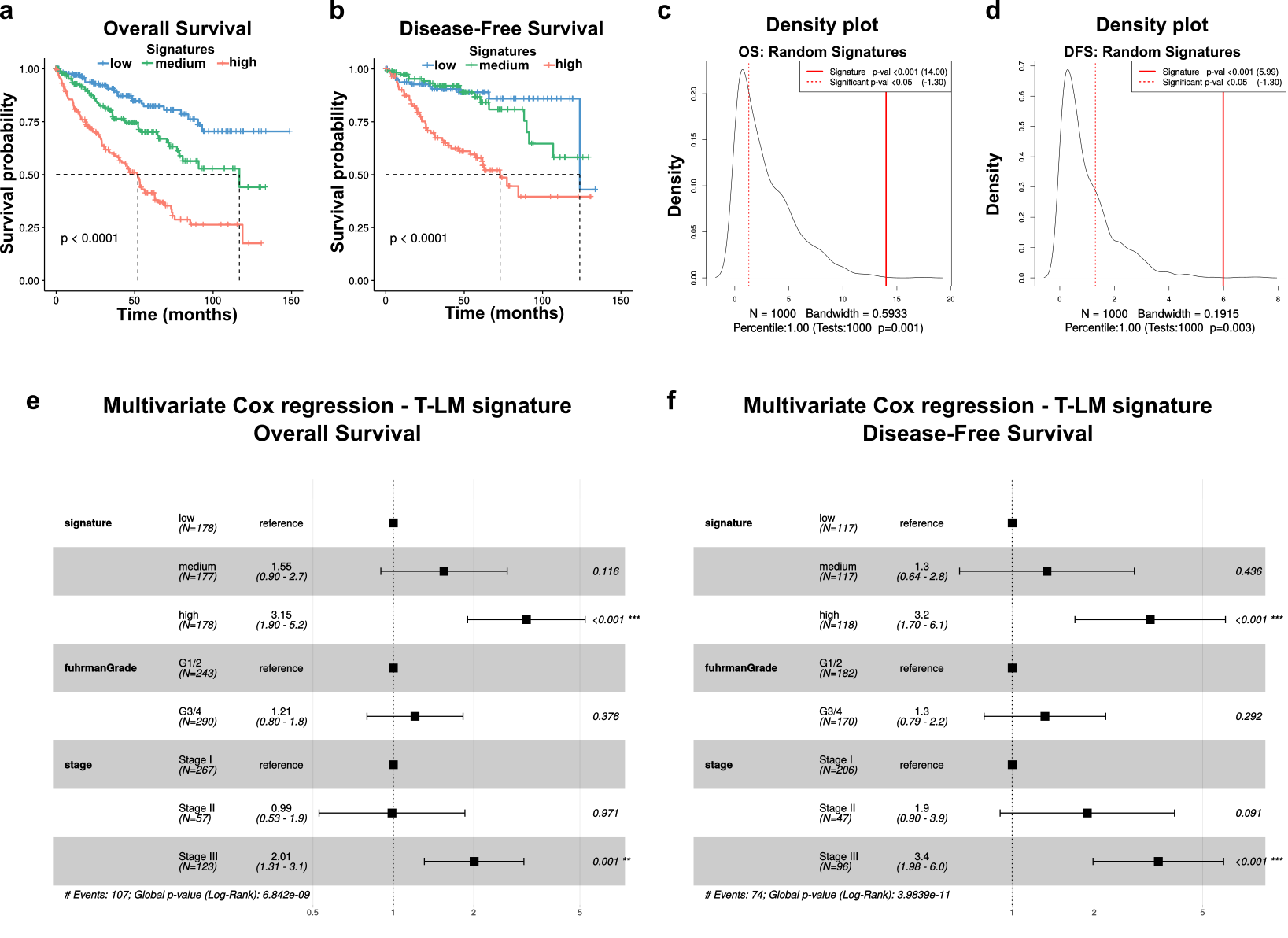

Supplement: Supplementary file 7 — Additional file 7: Supplementary Fig. S6. [file 12943_2021_1416_MOESM7_ESM.png]

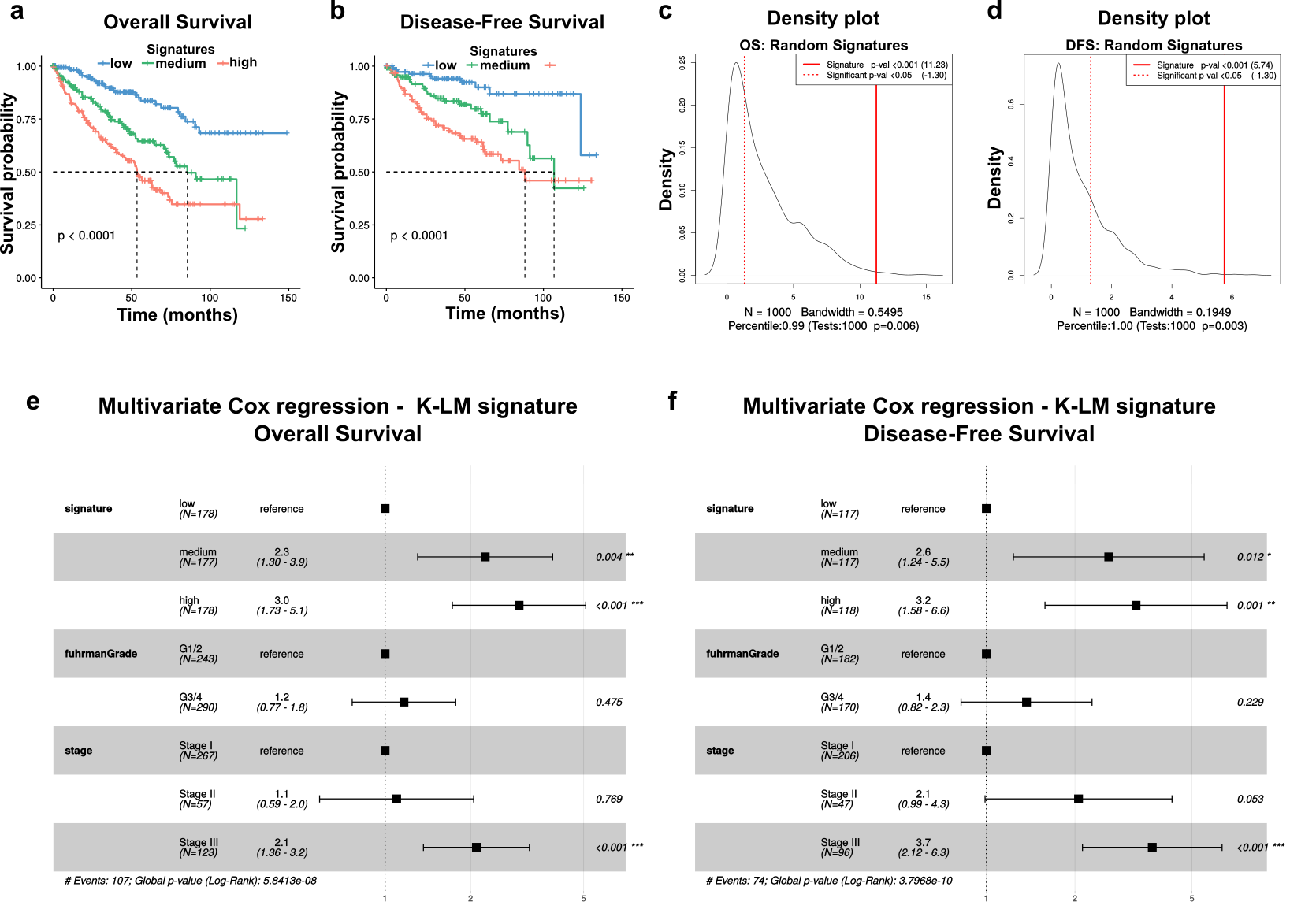

Supplement: Supplementary file 8 — Additional file 8: Supplementary Fig. S7. [file 12943_2021_1416_MOESM8_ESM.png]

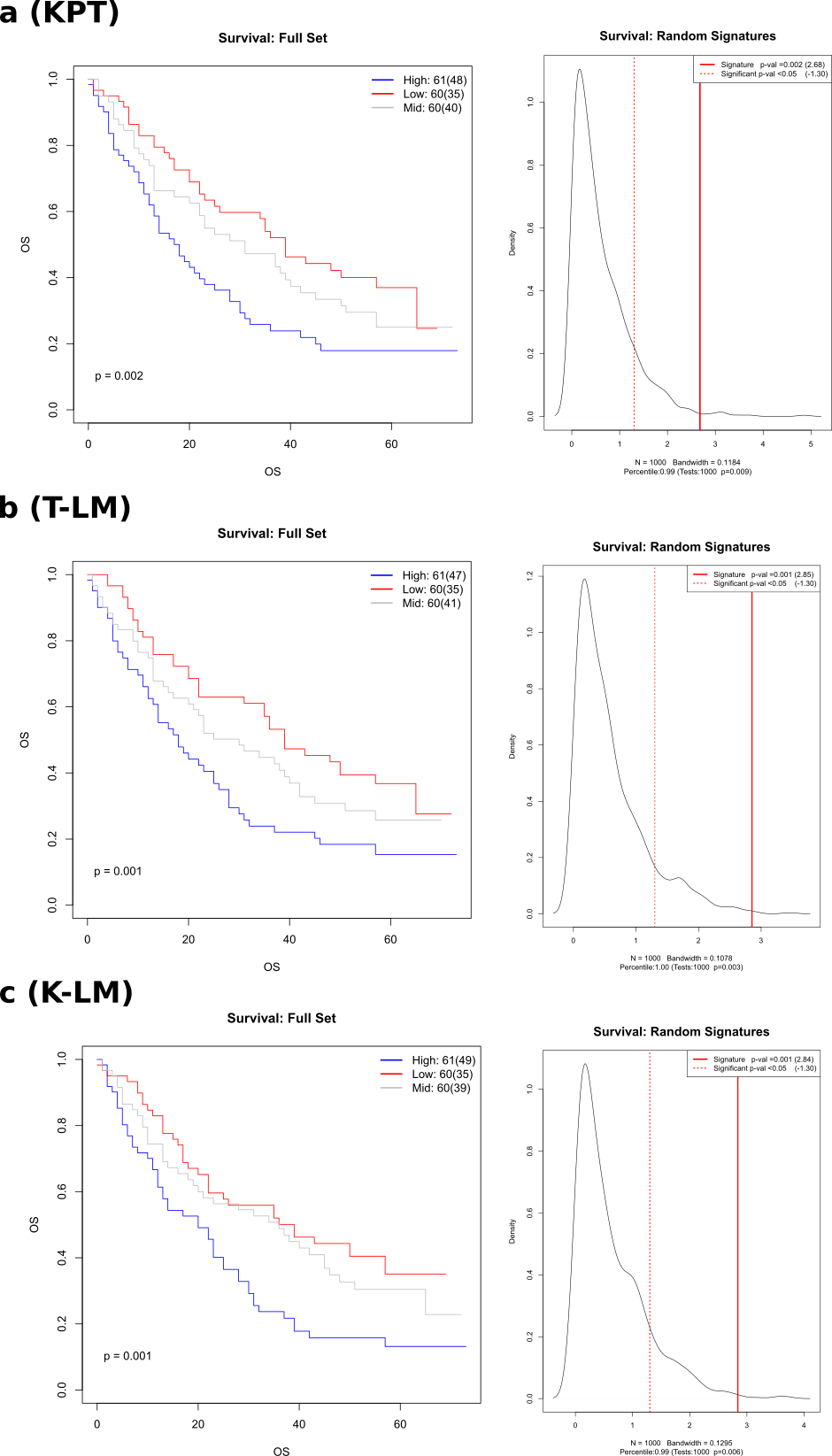

Supplement: Supplementary file 9 — Additional file 9: Supplementary Fig. S8. [file 12943_2021_1416_MOESM9_ESM.png]

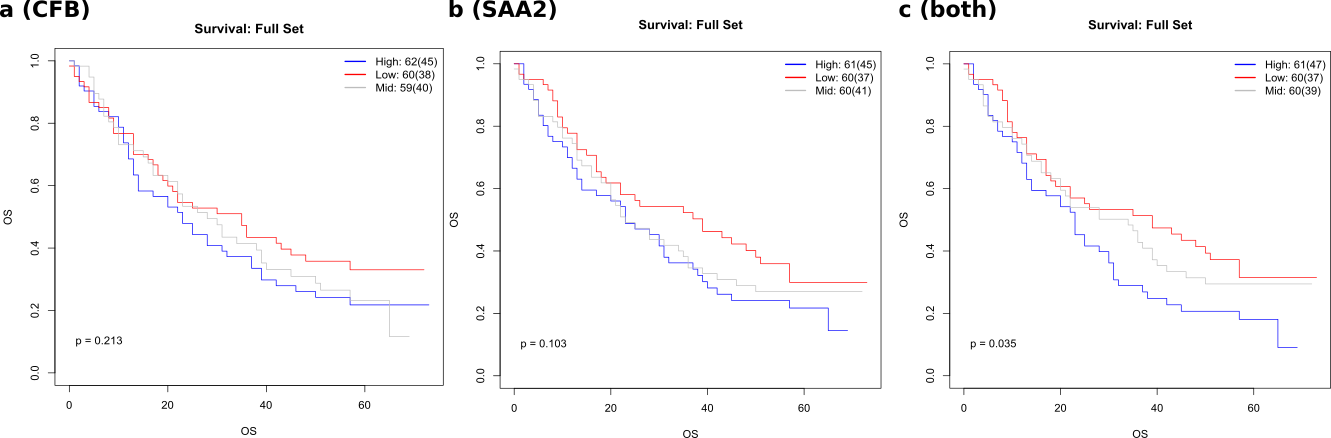

Supplement: Supplementary file 10 — Additional file 10: Supplementary Fig. S9. [file 12943_2021_1416_MOESM10_ESM.png]
